# Crystal structures of the recreational drug *N*-(4-meth­oxy­phen­yl)piperazine (MeOPP) and three of its salts

**DOI:** 10.1107/S2056989020002844

**Published:** 2020-03-05

**Authors:** Haruvegowda Kiran Kumar, Hemmige S. Yathirajan, Chayanna Harish Chinthal, Sabine Foro, Christopher Glidewell

**Affiliations:** a Department of Studies in Chemistry, University of Mysore, Manasagangotri, Mysuru 570 006, India; b Institute of Materials Science, Darmstadt University of Technology, Alarich-Weiss-Strasse 2, D-64287 Darmstadt, Germany; c School of Chemistry, University of St Andrews, St Andrews, Fife KY16 9ST, UK

**Keywords:** piperazines, crystal structure, mol­ecular dimensions, mol­ecular conformation, hydrogen bonding, supra­molecular assembly

## Abstract

Crystal structures are reported for *N*-(4-meth­oxy­phen­yl)piperazine (MeOPP), (I), and for its 3,5-di­nitro­benzoate, 2,4,6-tri­nitro­phenolate (picrate) and 4-amino­benzoate salts, (II)–(IV), the last of which crystallizes as a monohydrate. In MeOPP, C_11_H_16_N_2_O, (I), the 4-meth­oxy­phenyl group is nearly planar and it occupies an equatorial site on the piperazine ring: the mol­ecules are linked into simple *C*(10) chains by N—H⋯O hydrogen bonds. In each of the salts, *i.e.*, C_11_H_17_N_2_O^+^·C_7_H_3_N_2_O_6_^−^, (II), C_11_H_17_N_2_O^+^·C_6_H_2_N_3_O_7_^−^, (III), and C_11_H_17_N_2_O^+^·C_7_H_6_NO_2_^−^·H_2_O, (IV), the effectively planar 4-meth­oxy­phenyl substituent again occupies an equatorial site on the piperazine ring. In (II), two of the nitro groups are disordered over two sets of atomic sites and the bond distances in the anion indicate considerable delocalization of the negative charge over the C atoms of the ring. The ions in (II) are linked by two N—H⋯O hydrogen bonds to form a cyclic, centrosymmetric four-ion aggregate; those in (III) are linked by a combination of N—H⋯O and C—H⋯π(arene) hydrogen bonds to form sheets; and the components of (IV) are linked by N—H⋯O, O—H⋯O and C—H⋯π(arene) hydrogen bonds to form a three-dimensional framework structure. Comparisons are made with the structures of some related compounds.

## Abstract 2

The hydrogen-bonded assemblies in *N*-(4-meth­oxy­phen­yl)piperazine and its 3,5-di­nitro­benzoate, picrate, and monohydrated 4-amino­benzoate salts are, respectively, one-, zero-, two- and three-dimensional.

## Chemical context   

*N*-(4-Meth­oxy­phen­yl)piperazine (MeOPP) has fairly recently emerged as a new addition to the range of designer drugs aimed at recreational use, and considerable effort has consequently been invested in the development of rapid and reliable methods for the detection in human fluids not only of MeOPP itself but also of its primary metabolites *N*-(4-hy­droxy­phen­yl)piperazine and 4-hy­droxy­aniline (Staack & Maurer, 2003 ▸; Staack *et al.*, 2004 ▸). The action of MeOPP on human physiology is similar to that of amphetamines, but it has a significantly lower potential for abuse (Nagai *et al.*, 2007 ▸). In view of these observations, coupled with the broad range of biological activities exhibited by piperazine derivatives in general (Asif, 2015 ▸; Brito *et al.*, 2019 ▸), we have recently initiated a programme of study centred on *N*-(4-meth­oxy­phen­yl)piperazine derivatives. Thus, we have recently reported the synthesis and structures of a range of salts derived from MeOPP (Kiran Kumar, Yathirajan, Foro *et al.*, 2019 ▸), as well as those of a range of neutral 1-aroyl-4-(4-meth­oxy­phen­yl)piperazines (Kiran Kumar, Yathirajan, Sagar *et al.*, 2019 ▸). In a continuation of the earlier work, we have now prepared a further series of salts, whose mol­ecular and supra­molecular structures we report here, along with that of MeOPP itself: the structures reported here are those of *N*-(4-meth­oxy­phen­yl)piperazine (I), 4-(4-meth­oxy­phen­yl)pip­er­az­in-1-ium 3,5-di­nitro­benzoate (II), 4-(4-meth­oxy­phen­yl)piperazin-1-ium 2,4,6-tri­nitro­phenolate (III) and 4-(4-meth­oxy­phen­yl)piperazin-1-ium 4-amino­benzoate monohydrate (IV) (Figs.  ▸1–4 ▸ ▸ ▸). The salts (II)–(IV) were readily prepared by co-crystallization of MeOPP with the appropriate acidic component in methanol.
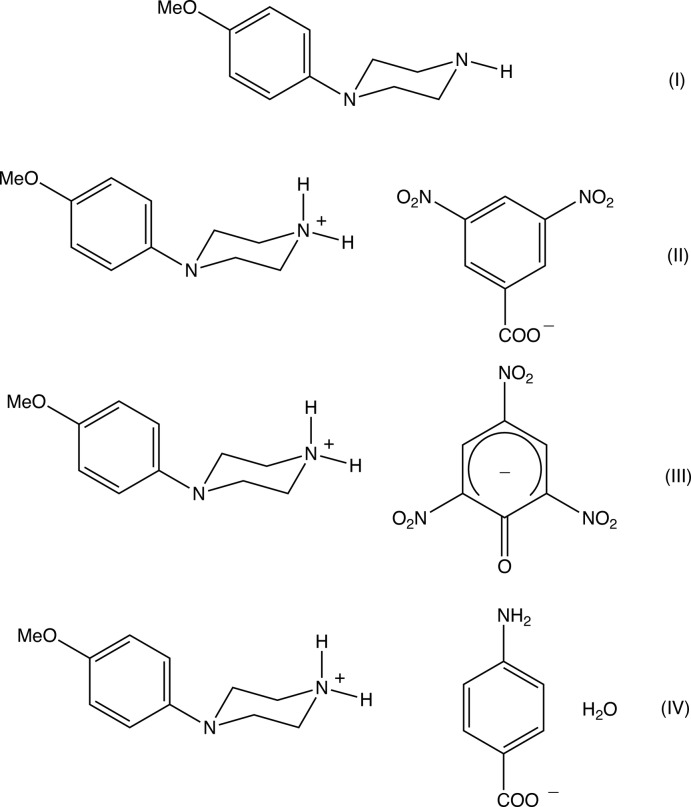


## Structural commentary   

Compound (I) is the neutral *N*-(4-meth­oxy­phen­yl)piperazine (MeOPP), and compounds (II) and (III) are unsolvated 1:1 3,5-di­nitro­benzoate and 2,4,6-tri­nitro­phenolate (picrate) salts, respectively, while compound (IV) is the 1:1 4-amino­benzoate salt, which crystallizes as a stoichiometric monohydrate in which the water component is firmly embedded in the overall hydrogen-bonded network (see Section 3, below). In each of (I)–(IV), the 4-meth­oxy­phenyl substituent occupies an equatorial site on the piperazine ring but the MeOPP component exhibits no inter­nal symmetry, so that it is conformationally chiral: the space groups (Table 2 ▸) confirm that each compound has crystallized as a conformational racemate. In each compound, the reference MeOPP unit was selected as one having a torsional angle C23—C24—O24—C27 that was close to 180°, as opposed to the alternative value close to zero degrees, and with the ring-puckering angle θ (Cremer & Pople, 1975 ▸), as calculated for the atom sequence (N1,C2,C3,N4,C5,C6) which was close to 0°, as opposed to a value close to 180° for the opposite conformational enanti­omer.

**Figure 3:**
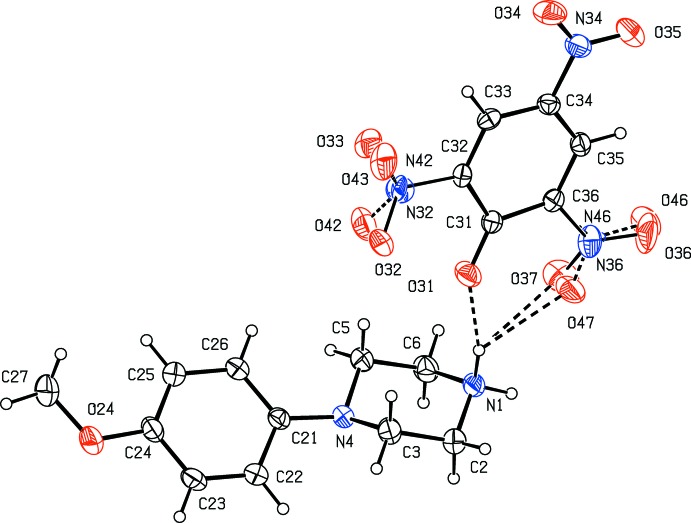
The independent components of compound (III) showing the atom-labelling scheme, the hydrogen bonds, drawn as dashed lines, within the selected asymmetric unit, and the disorder in the nitro groups: the major disorder components are drawn with full lines and the minor disorder components are drawn with broken lines. Displacement ellipsoids are drawn at the 30% probability level.

In the salt (III), the nitro substituents at atoms C32 and C36 (Fig. 3 ▸) are both disordered over two sets of atomic sites having refined occupancies of 0.531 (16) and 0.469 (16) for the nitro group at atom C32, and 0.62 (6) and 0.38 (6) for that at atom C36. The major and minor disorder components of both these nitro groups are rotated about the exocyclic C—N bonds: for the C32 substituent, the two components are rotated by similar amounts, 22.6 (5) and 24.2 (5)° for the major and minor components, but in opposite senses, so that the dihedral angle between the two components is 46.8 (6)°; by contrast, the rotations at C36 are in the same sense, by 25.2 (8) and 5.0 (3)°, with a dihedral angle between the components of 20.7 (18)°. The bond distances within this anion show some inter­esting features: firstly, the distance C31—O31, 1.235 (2) Å, is short for a phenolic bond [mean value (Allen *et al.*, 1987 ▸) 1.362 Å, lower quartile value 1.353 Å] and more reminiscent of the distances observed in ketones (mean value 1.210 Å); secondly, the two C—C distances flanking this C—O unit, 1.448 (3) and 1.455 (3) Å, are much longer that the other C—C distances in this ring, which lie in the range 1.364 (3)–1.383 (3) Å. These metrical observations support the formulation of the picrate anion here as containing an effectively double C=O bond at atom C31, with extensive delocalization of the negative charge over the atoms C32–C36, as indicated in the scheme.

In each compound, the meth­oxy C atom lies close to the plane of the adjacent aryl ring: the deviations from this plane are 0.176 (5), 0.033 (3), 0.040 (6) and 0.277 (7) Å in (I)–(IV), respectively. Associated with this near co-planarity, the two exocyclic O—C—C angles differ by *ca* 10° in each case, as is often observed when alk­oxy­arene systems are planar or nearly so (Seip & Seip, 1973 ▸; Ferguson *et al.*, 1996 ▸).

## Supra­molecular features   

**Figure 5:**
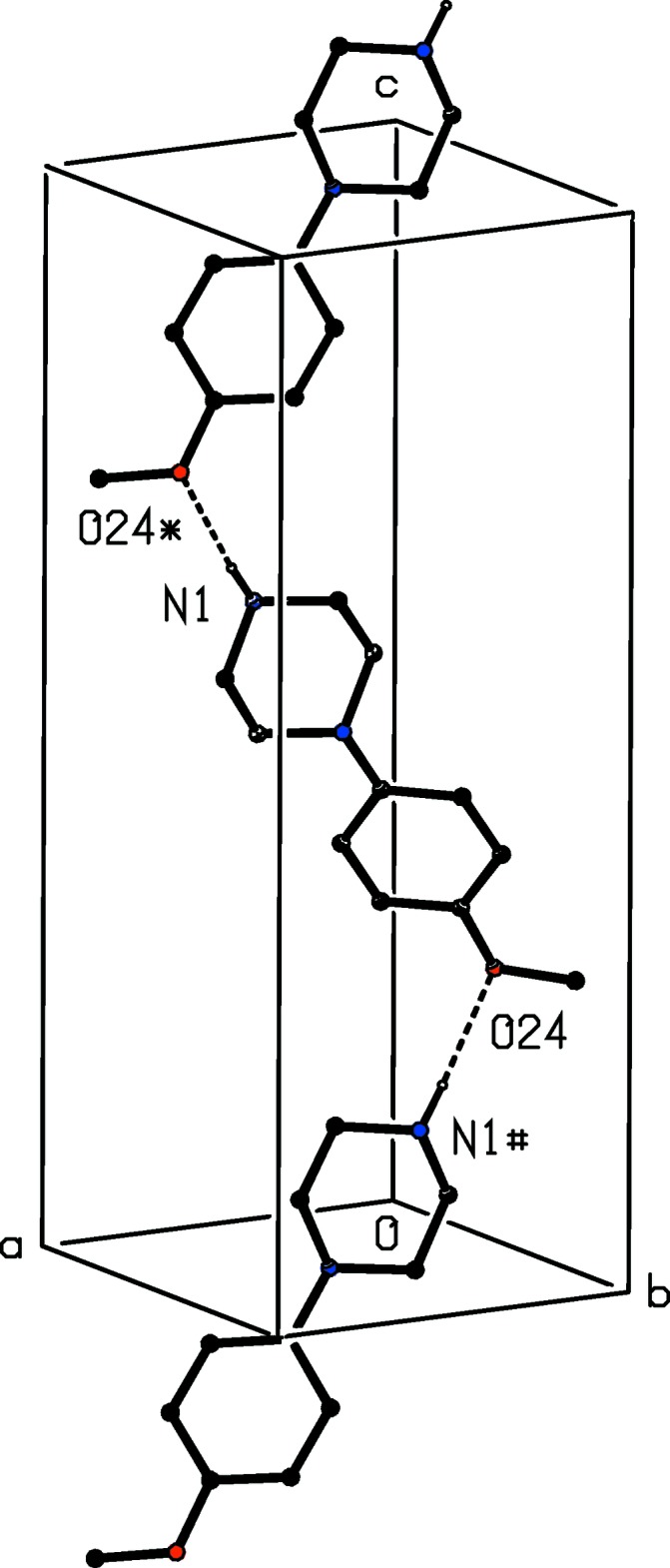
Part of the crystal structure of compound (I) showing the formation of a hydrogen-bonded chain parallel to [001]. Hydrogen bonds are drawn as dashed lines and, for the sake of clarity, the H atoms bonded to C atoms have been omitted. The atoms marked with an asterisk (*) or a hash (#) are at the symmetry positions (1 − *x*, 1 − *y*, 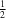 + *z*) and (1 − *x*, 1 − *y*, −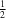 + *z*), respectively.

The supra­molecular assembly of compound (I) is extremely simple: a single N—H⋯O hydrogen bond (Table 1 ▸) links mol­ecules that are related by a 2_1_ screw axis to form a *C*(10) (Etter, 1990 ▸; Etter *et al.*, 1990 ▸; Bernstein *et al.*, 1995 ▸) chain running parallel to the [001] direction (Fig. 5 ▸). However, there are no direction-specific inter­actions between adjacent chains so that the supra­molecular assembly here is one-dimensional.

**Figure 6:**
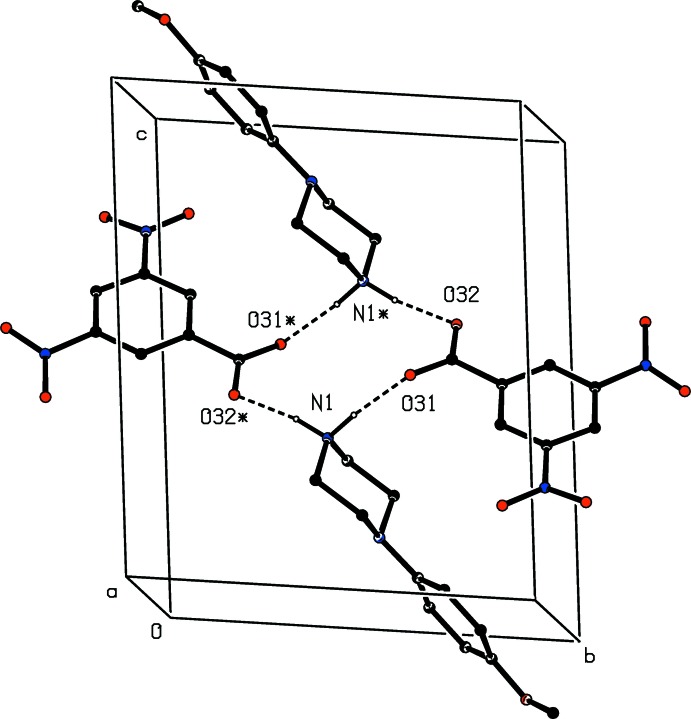
Part of the crystal structure of compound (II) showing the formation of a cyclic hydrogen-bonded 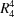
(12) aggregate. Hydrogen bonds are drawn as dashed lines and, for the sake of clarity, the H atoms bonded to C atoms have been omitted. The atoms marked with an asterisk (*) are at the symmetry position (1 − *x*, 1 − *y*, 1 − *z*).

The assembly in the 3,5-di­nitro­benzoate salt (II) is also very simple. Two independent N—H⋯O hydrogen bonds (Table 1 ▸) link inversion-related ion-pairs to form a cyclic centrosymmetric four-ion aggregate characterized by an 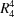
(12) motif, and centred at (0.5, 0.5, 0.5) (Fig. 6 ▸). There are no direction-specific inter­actions between adjacent aggregates, so that the supra­molecular assembly here is finite, or zero-dimensional.

**Figure 7:**
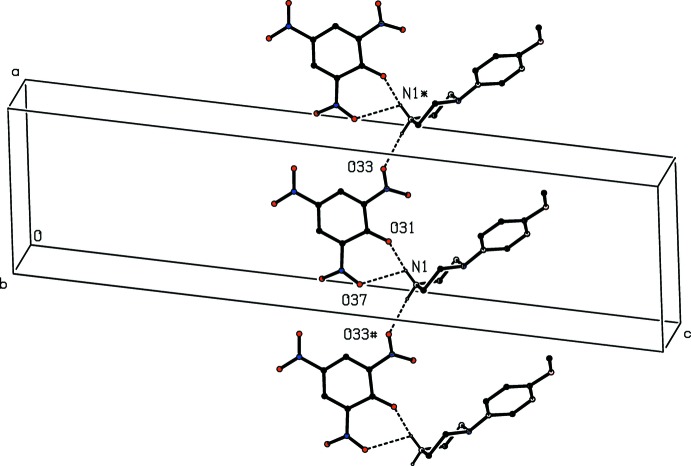
Part of the crystal structure of compound (III) showing the formation of a hydrogen-bonded chain of rings parallel to [100]. Hydrogen bonds are drawn as dashed lines and, for the sake of clarity, the minor disorder components and the H atoms bonded to C atoms have been omitted. The atoms marked with an asterisk (*) or a hash (#) are at the symmetry positions (1 + *x*, *y*, *z*) and (−1 + *x*, *y*, *z*), respectively.

**Figure 8:**
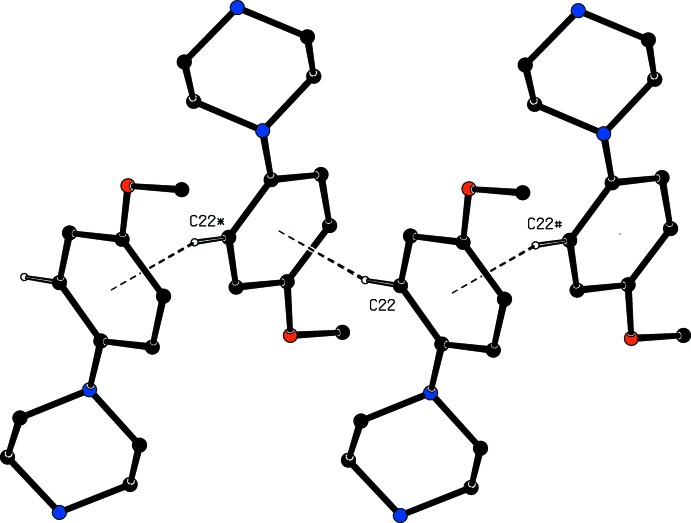
Part of the crystal structure of compound (III), showing the formation of a hydrogen-bonded chain of cations along [010]. Hydrogen bonds are drawn as dashed lines and, for the sake of clarity, the unit-cell outline, the minor disorder components and the H atoms not involved in the motif shown have been omitted. The atoms marked with an asterisk (*) or a hash (#) are at the symmetry positions (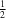 − *x*, −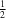 + *y*, 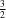 − *z*) and (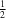 − *x*, 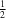 + *y*, 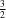 − *z*), respectively.

**Figure 9:**
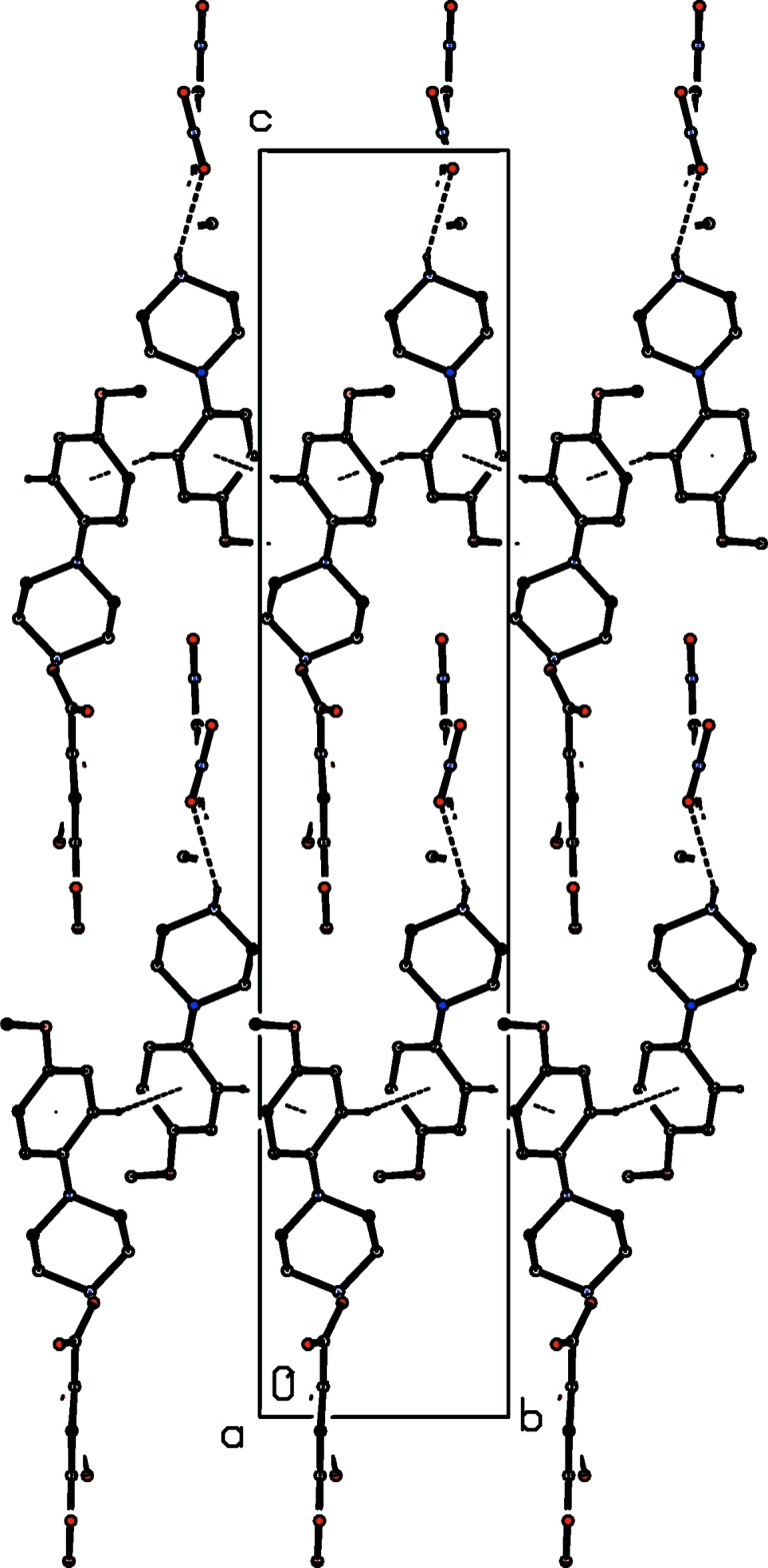
A projection along [100] of part of the crystal structure of (III), showing the inter­digitation of the sheets lying parallel to (001). Hydrogen bonds are drawn as dashed lines and, for the sake of clarity, the minor disorder components and the H atoms not involved in the motifs shown have been omitted.

The component ions of compound (III) are linked by a combination of N—H⋯O and C—H⋯π(arene) hydrogen bonds to form complex sheets; however, the formation of the sheet structure is readily analysed in terms of two simpler, one-dimensional sub-structures (Ferguson *et al.*, 1998*a* ▸,**b* ▸;* Gregson *et al.*, 2000 ▸). Although two of the nitro groups exhibit positional disorder (see Section 2, above), the hydrogen bonds involving the two sets of disorder components are fairly similar (Table 1 ▸), so that it is only necessary here to consider the inter­actions involving the major disorder components. The two ions within the selected asymmetric unit (Fig. 3 ▸) are linked by a markedly asymmetric N—H⋯(O)_2_ three-centre system containing an 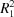
(6) ring, and ion-pairs of this type, which are related by translation, are linked by a two-centre N—H⋯O hydrogen bond to form a *C*(8)*C*(11)[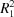
(6)] chain of rings running parallel to [100] (Fig. 7 ▸). In the second sub-structure, cations, which are related by a 2_1_ screw axis, are linked by a C—H⋯π(arene) hydrogen bond, to form a chain running parallel to the [010] direction (Fig. 8 ▸). The combination of chains running parallel to the [100] and [010] directions then generates a sheet lying parallel to (001) in the domain 0.5 < z < 1.0. A second sheet of this type, related to the first by inversion, lies in the domain 0 < z < 0.5: although there are no direction-specific inter­actions between adjacent sheets, so that the supra­molecular assembly in (III) is two dimensional, the sheets are, however, strongly inter­digitated (Fig. 9 ▸).

**Figure 10:**
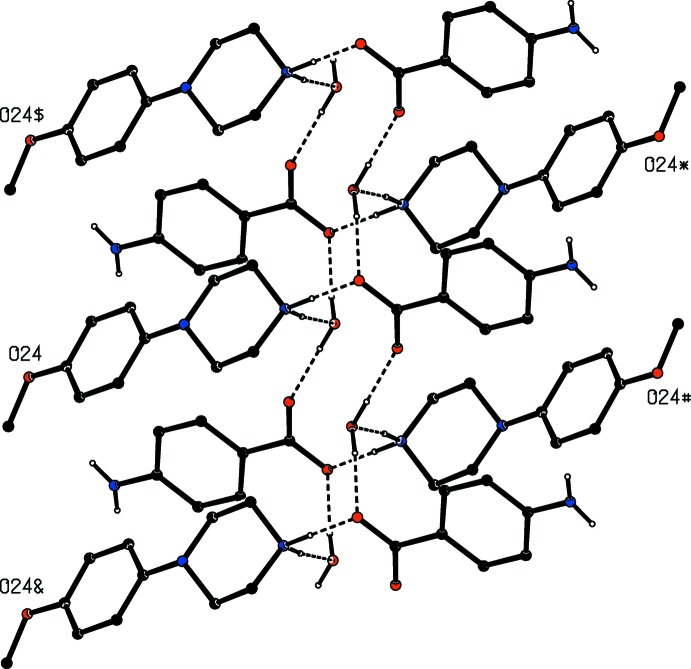
Part of the crystal structure of compound (IV) showing the formation of a hydrogen-bonded chain of rings parallel to [100]. Hydrogen bonds are drawn as dashed lines and, for the sake of clarity, the unit-cell outline and the H atoms bonded to C atoms have been omitted. The atoms marked with an asterisk (*), a hash (#), a dollar sign ($) or an ampersand (&) are at the symmetry positions (1 − *x*, 1 − *y*, 2 − *z*), (−*x*, 1 − *y*, 2 − *z*), (1 + *x*, *y*, *z*) and (−1 + *x*, *y*, *z*) respectively.

**Figure 11:**
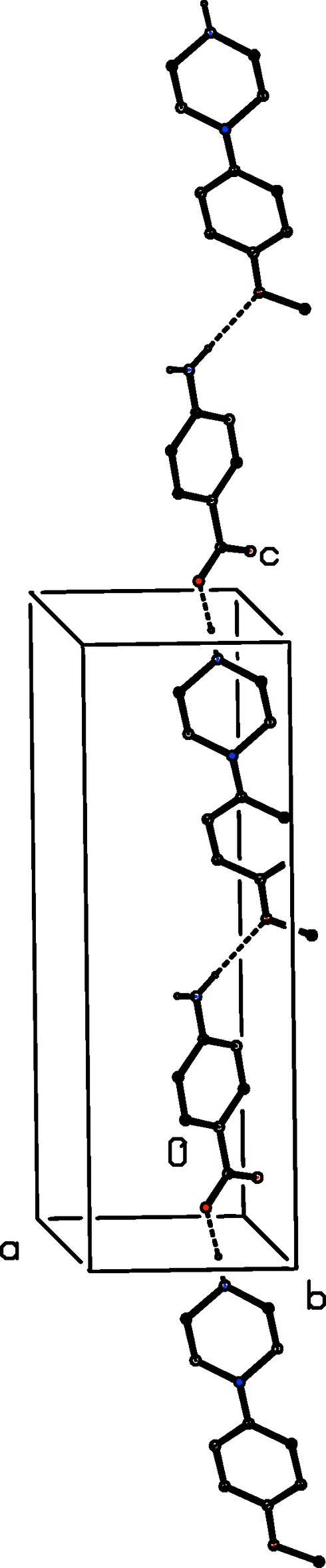
Part of the crystal structure of compound (IV) showing the formation of a hydrogen-bonded chain parallel to [001]. Hydrogen bonds are drawn as dashed lines and, for the sake of clarity, the water mol­ecules and the H atoms bonded to C atoms have been omitted.

For compound (IV), the supra­molecular assembly is more complex than for any of compounds (I)–(III), as a result of the presence of both an additional amino substituent in the cation and a water mol­ecule, which acts as both a donor and an acceptor of hydrogen bonds (Table 1 ▸). The combination of N—H⋯O, O—H⋯O and C—H⋯π(arene) hydrogen bonds links the components into a three-dimensional framework structure but, again, this can readily be analysed in terms of fairly simple sub-structures. In the first of these, the ionic components and the water mol­ecules form a chain of centrosymmetric rings running parallel to the [100] direction, in which 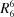
(16) rings centred at (*n*, 0.5, 1) alternate with 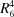
(12) rings centred at (*n* + 0.5, 0.5, 1), where *n* represents an integer in each case (Fig. 10 ▸). In the second sub-structure, the two N—H⋯O hydrogen bonds having atoms O24 and O31 as the acceptors (Table 1 ▸) link the ions into a simple 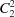
(18) chain running parallel to the [001] direction (Fig. 11 ▸).

**Figure 12:**
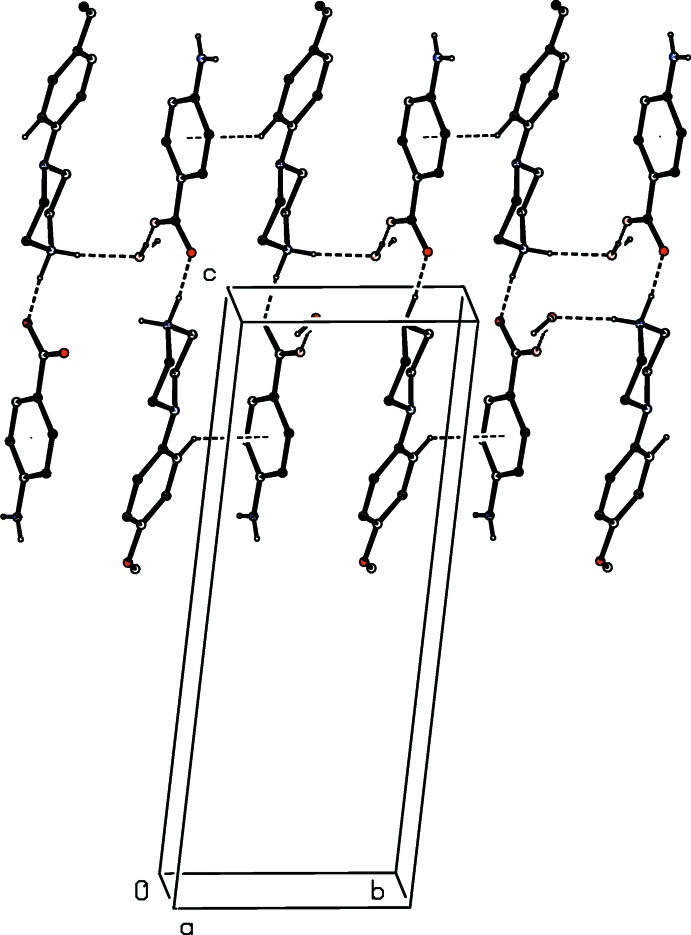
Part of the crystal structure of compound (IV) showing the formation of a hydrogen-bonded chain parallel to [010] and built from N—H⋯O, O—H⋯O and C—H⋯π(arene) hydrogen bonds; these are drawn as dashed lines and, for the sake of clarity, the H atoms bonded to the C atoms not involved in the motifs shown have been omitted.

There are two C—H⋯π(arene) inter­actions in the structure of compound (IV): the longer of these, involving atom C22, lies within the chain of rings along [100] but the other, shorter, inter­action combines with some of the N—H⋯O and O—H⋯O hydrogen bonds to generate a complex chain running parallel to the [010] direction (Fig. 12 ▸). The combination of chains along [100], [010] and [001] then suffices to generate a three-dimensional supra­molecular structure.

Hence the supra­molecular aggregation is zero-, one-, two- and three-dimensional in compounds (II), (I), (III) and (IV), respectively.

## Database survey   

The first salt of MeOPP to have its structure reported was 4-(4-meth­oxy­phen­yl)piperazin-1-ium chloride (V) (Zia-ur-Rehman *et al.*, 2009 ▸), in which two N—H⋯Cl hydrogen bonds link the ions into simple chains. The aggregation in the 3,5-di­nitro­benzoate salt (II) reported here, where two independent N—H⋯O hydrogen bonds generate a cyclic 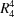
(12) motif, can be contrasted with that in the tri­chloro­acetate salt (VI) (Kiran Kumar, Yathirajan, Foro *et al.*, 2019 ▸), where two independent N—H⋯O hydrogen bonds generate a continuous 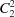
(6) chain: the reason for the finite aggregation in (II) *versus* the continuous aggregation in (VI) is not obvious. The electronic delocalization in the anion of (III) reported here is similar to that in the anion of the 5-hy­droxy-3,5-di­nitro­benzoate salt (VII) (Kiran Kumar, Yathirajan, Foro *et al.*, 2019 ▸), where it is the phenolic hydroxyl group that has ionized rather than the carboxyl group, so forming an anion more reminiscent of a picrate ion than of a 3,5-di­nitro­benzoate ion. The aggregation in (VII) takes the form of a chain of rings generated by a combination of N—H⋯O and C—H⋯O hydrogen bonds, with chains of this type further linked by C—H⋯π(arene) hydrogen bonds to form a three-dimensional structure. The unit-cell dimensions of compound (IV) reported here are similar to those in a series of isomorphous monohydrated benzoate salts containing anions of type (4–C_6_H_4_COO)^−^, where - = H, F, Cl or Br, compounds (VIII)–(XI), in all of which the 4-meth­oxy­phenyl unit exhibits disorder (Kiran Kumar, Yathirajan, Foro *et al.*, 2019 ▸): however, despite the similarity in cell dimensions, the structure of (IV) differs from those of (VIII)–(XI) firstly in showing no disorder and secondly in forming a three-dimensional hydrogen-bonded structure as opposed to the one-dimensional assembly in (VIII)–(XI). By contrast with compounds (VIII)–(XI) in space group *P* 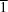, the 4-iodo­benzoate analogue (XII), also a monohydrate (Kiran Kumar, Yathirajan, Harish Chinthal *et al.*, 2020 ▸) crystallizes in space group *P*2_1_/*c* with *Z*′ = 3, but with no disorder, and an extensive series of N—H⋯O and O—H⋯O hydrogen bonds links the nine independent components into complex sheets.

## Synthesis and crystallization   

*N*-[4-Meth­oxy­phen­yl]piperazine (I), was purchased from Sigma–Aldrich, and crystals suitable for single-crystal X-ray diffraction were grown by slow evaporation, at ambient temperature and in the presence of air, of a solution in methanol, m.p. 316–318 K. For the preparation of the salts (II)–(IV), solutions of (I) (100 mg, 0.52 mmol) in methanol (10 ml), and of 0.52 mmol of the appropriate acidic component [3,5-di­nitro­benzoic acid (110.3 mg) for (II), picric acid (119.1 mg) for (III), and 4-amino­benzoic acid (71.3 mg) for (IV)] also in methanol (10 ml) were mixed and then briefly held at 313 K with stirring. The solutions were allowed to cool to ambient temperature and then set aside to crystallize, giving the products (II)–(IV). The products were collected by filtration, and dried in air: m.p. (II) 393–395 K, (III) 420–422 K, and (IV) 407–409 K. Crystals of the salts (II)–(IV) suitable for single-crystal X-ray diffraction were grown by slow evaporation, at ambient temperature and in the presence of air, of solutions in methanol/ethyl acetate (1:1, *v*/*v*).

## Refinement   

Crystal data, data collection and refinement details are summarized in Table 2 ▸. All H atoms were located in difference maps. The H atoms bonded to C atoms were then treated as riding atoms in geometrically idealized positions with C—H distances of 0.93 Å (aromatic), 0.96 Å (CH_3_) or 0.97 Å (CH_2_), and with *U*_iso_(H) = *kU*_eq_(C), where *k* = 1.5 for the methyl groups which were permitted to rotate but not to tilt, and 1.2 for all other H atoms bonded to C atoms. For the H atoms bonded to N atoms in (I) and (II), the atomic coordinates were refined with *U*_iso_(H) = 1.2*U*_eq_(N) giving the N—H distances shown in Table 1 ▸. In (III) and (IV), free refinement of the atomic coordinates for the H atoms bonded to N in the cations, and to O in the water component of (IV) gave N—H and O—H distances which were rather unsatisfactory: hence these H atoms bonded to N were treated as riding atoms with *U*_iso_(H) = 1.2*U*_eq_(N), while for those bonded to O in (IV), the O—H distances were restrained to a value of 0.84 (2) Å, with *U*_iso_(H) = 1.5*U*_eq_(O), giving the distances shown in Table 1 ▸. In compound (III), two of the nitro groups exhibited disorder over two sets of atomic sites having unequal occupancy. For the minor disorder components, the bonded distances and the 1,3 non-bonded distances were restrained to be the same as the corresponding distances in the major disorder components subject to s. u. values of 0.01 and 0.02 Å, respectively, giving refined occupancies of 0.531 (16) and 0.469 (16) for the nitro group at atom C32, and 0.62 (6) and 0.38 (6) for that at atom C36. In addition, for each of the disordered substituents, the component atoms were restrained to have the same *U^ij^* components. In the absence of significant resonant scattering, the correct orientation of the structure of (I) with respect to the polar axis direction could not be established: the value of the Flack *x* parameter (Flack, 1983 ▸), as calculated (Parsons *et al.*, 2013 ▸) using 546 quotients of the type [(*I* ^+^) − (*I* ^−^)]/[(*I* ^+^) + (*I* ^−^)] was −0.5 (8), so that the correct orientation is indeterminate (Flack & Bernardinelli, 2000 ▸): however, in the space group *Pna*2_1_, this parameter does not carry any information of chemical significance.

## Supplementary Material

Crystal structure: contains datablock(s) global, I, II, III, IV. DOI: 10.1107/S2056989020002844/zl2772sup1.cif

Structure factors: contains datablock(s) I. DOI: 10.1107/S2056989020002844/zl2772Isup2.hkl

Structure factors: contains datablock(s) II. DOI: 10.1107/S2056989020002844/zl2772IIsup3.hkl

Structure factors: contains datablock(s) III. DOI: 10.1107/S2056989020002844/zl2772IIIsup4.hkl

Structure factors: contains datablock(s) IV. DOI: 10.1107/S2056989020002844/zl2772IVsup5.hkl

Supporting information file. DOI: 10.1107/S2056989020002844/zl2772Isup6.cml

Supporting information file. DOI: 10.1107/S2056989020002844/zl2772IIsup7.cml

Supporting information file. DOI: 10.1107/S2056989020002844/zl2772IIIsup8.cml

Supporting information file. DOI: 10.1107/S2056989020002844/zl2772IVsup9.cml

CCDC references: 1987252, 1987251, 1987250, 1987249

Additional supporting information:  crystallographic information; 3D view; checkCIF report

## Figures and Tables

**Figure 1:**
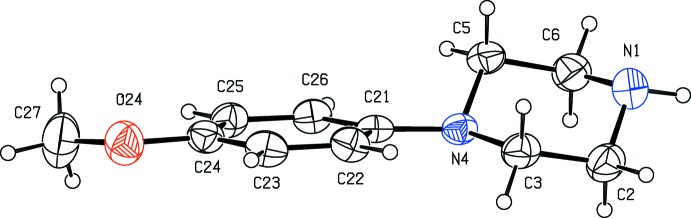
The mol­ecular structure of compound (I) showing the atom-labelling scheme. Displacement ellipsoids are drawn at the 30% probability level.

**Figure 2:**
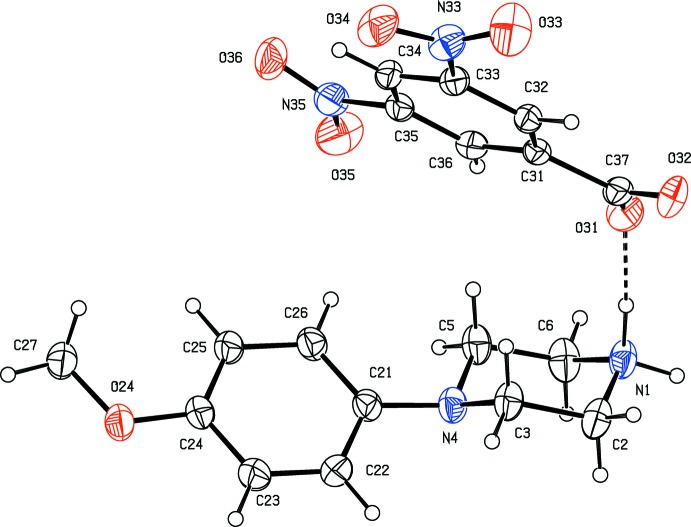
The independent components of compound (II) showing the atom-labelling scheme and the hydrogen bond, drawn as a dashed line, within the selected asymmetric unit. Displacement ellipsoids are drawn at the 30% probability level.

**Figure 4:**
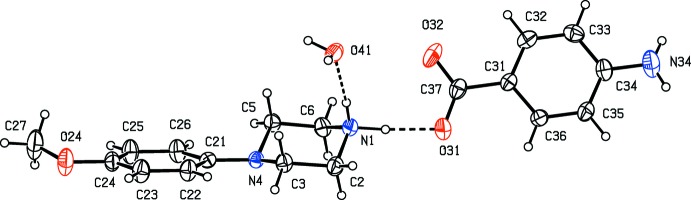
The independent components of compound (IV) showing the atom-labelling scheme and the hydrogen bonds, drawn as dashed lines, within the selected asymmetric unit. Displacement ellipsoids are drawn at the 30% probability level.

**Table 1:** Hydrogen bonds and short inter­molecular contacts (Å, °) for compounds (I)–(IV) *Cg*1 and *Cg*2 represent the centroids of the rings (C21–C26) and (C31–C36), respectively.

Compound	*D*—H⋯*A*	*D*—H	H⋯*A*	*D*⋯*A*	*D*—H⋯*A*
(I)	N1—H1⋯O24^i^	0.91 (4)	2.23 (4)	3.139 (3)	171 (3)
(II)	N1—H11⋯O31	0.96 (2)	1.814 (19)	2.7638 (18)	169 (2)
	N1—H12⋯O32^ii^	0.964 (18)	1.740 (18)	2.6953 (18)	170.5 (17)
(III)	N1—H11⋯O31	0.92 (3)	1.81 (3)	2.704 (3)	163 (2)
	N1—H11⋯O37	0.92 (3)	2.56 (3)	2.982 (11)	108.7 (19)
	N1—H11⋯O47	0.92 (3)	2.42 (3)	2.870 (15)	110.1 (19)
	N1—H12⋯O33^iii^	0.91 (3)	2.12 (3)	2.926 (6)	148 (2)
	N1—H12⋯O43^iii^	0.91 (3)	1.92 (3)	2.815 (6)	168 (2)
	N1—H12⋯O47	0.91 (3)	2.54 (3)	2.870 (15)	102.1 (19)
	C12—H12⋯*Cg*1^iv^	0.93	2.86	3.769 (3)	164
(IV)	N1—H11⋯O41	0.95 (2)	1.88 (2)	2.803 (3)	165.2 (8)
	N1—H12⋯O31	0.943 (7)	1.793 (18)	2.728 (3)	171.2 (7)
	O41—H41⋯O32^v^	0.85 (3)	1.78 (3)	2.631 (4)	178 (4)
	O41—H42⋯O31^vi^	0.85 (3)	1.95 (3)	2.772 (3)	164 (3)
	N34—H341⋯O24^vii^	0.82 (4)	2.23 (4)	3.057 (4)	177 (4)
	C22—H22⋯*Cg*2^v^	0.93	2.93	3.666 (3)	137
	C26—H26⋯*Cg*2^viii^	0.93	2.77	3.531 (3)	139

Symmetry codes: (i) 1 − *x*, 1 − *y*, 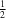 + *z*; (ii) 1 − *x*, 1 − *y*, 1 − *z*; (iii) −1 + *x*, *y*, *z*; (iv) 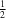 − *x*, −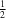 + *y*, 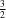 − *z*; (v) −*x*, 1 − *y*, 2 − *z*; (vi) 1 − *x*, 1 − *y*, 2 − *z*; (vii) *x*, *y*, 1 + *z*; (viii) −*x*, 2 − *y*, 2 − *z*.

**Table 2:** Experimental details

	(I)	(II)	(III)	(IV)
Crystal data				
Chemical formula	C_11_H_16_N_2_O	C_11_H_17_N_2_O^+^·C_7_H_3_N_2_O_6_^−^	C_11_H_17_N_2_O^+^·C_6_H_2_N_3_O_7_^−^	C_7_H_6_NO_2_^+^·C_11_H_17_N_2_O^−^·H_2_O
*M*_r_	192.26	404.38	421.37	347.41
Crystal system, space group	Orthorhombic, *P* *n* *a*2_1_	Triclinic, *P* 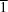	Monoclinic, *P*2_1_/*n*	Triclinic, *P* 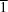
Temperature (K)	293	293	293	293
*a*, *b*, *c* (Å)	6.9683 (7), 7.9683 (8), 18.975 (2)	7.4365 (4), 10.6276 (6), 13.2700 (6)	8.7568 (6), 6.6292 (5), 34.024 (2)	6.2590 (7), 7.4549 (9), 19.269 (2)
α, β, γ (°)	90, 90, 90	92.238 (4), 97.057 (4), 108.618 (5)	90, 96.987 (6), 90	83.28 (1), 84.740 (1), 85.38 (1)
*V* (Å^3^)	1053.60 (19)	982.92 (9)	1960.4 (2)	886.94 (17)
*Z*	4	2	4	2
Radiation type	Mo *K*α	Mo *K*α	Mo *K*α	Mo *K*α
μ (mm^−1^)	0.08	0.11	0.12	0.09
Crystal size (mm)	0.48 × 0.48 × 0.40	0.50 × 0.48 × 0.48	0.48 × 0.42 × 0.20	0.40 × 0.20 × 0.14

Data collection				
Diffractometer	Oxford Diffraction Xcalibur with Sapphire CCD	Oxford Diffraction Xcalibur with Sapphire CCD	Oxford Diffraction Xcalibur with Sapphire CCD	Oxford Diffraction Xcalibur with Sapphire CCD
Absorption correction	Multi-scan (*CrysAlis RED*; Oxford Diffraction, 2009 ▸)	Multi-scan (*CrysAlis RED*; Oxford Diffraction, 2009 ▸)	Multi-scan (*CrysAlis RED*; Oxford Diffraction, 2009 ▸)	Multi-scan (*CrysAlis RED*; Oxford Diffraction, 2009 ▸)
*T*_min_, *T*_max_	0.814, 0.969	0.765, 0.950	0.844, 0.977	0.814, 0.987
No. of measured, independent and observed [*I* > 2σ(*I*)] reflections	4066, 1984, 1545	7013, 4202, 3057	14483, 4353, 2844	5786, 3500, 1923
*R*_int_	0.012	0.011	0.023	0.031
(sin θ/λ)_max_ (Å^−1^)	0.654	0.650	0.659	0.618

Refinement				
*R*[*F* ^2^ > 2σ(*F* ^2^)], *wR*(*F* ^2^), *S*	0.036, 0.088, 1.06	0.041, 0.110, 1.02	0.058, 0.136, 1.09	0.069, 0.187, 1.07
No. of reflections	1984	4202	4353	3500
No. of parameters	131	268	333	240
No. of restraints	1	0	216	2
H-atom treatment	H atoms treated by a mixture of independent and constrained refinement	H atoms treated by a mixture of independent and constrained refinement	H atoms treated by a mixture of independent and constrained refinement	H atoms treated by a mixture of independent and constrained refinement
Δρ_max_, Δρ_min_ (e Å^−3^)	0.10, −0.12	0.15, −0.19	0.18, −0.18	0.24, −0.20
Absolute structure	Flack x determined using 546 quotients [(*I* ^+^)−(*I* ^−^)]/[(*I* ^+^)+(*I* ^−^)] (Parsons *et al.*, 2013 ▸)	–	–	–

Computer programs: *CrysAlis CCD* and *CrysAlis RED* (Oxford Diffraction, 2009 ▸), *SHELXT* (Sheldrick, 2015*a* ▸), *SHELXL2014* (Sheldrick, 2015*b* ▸) and *PLATON* (Spek, 2020 ▸).

## supplementary crystallographic information

### N-(4-Methoxyphenyl)piperazine (I) Crystal data

**Table:**

C_11_H_16_N_2_O	*D*_x_ = 1.212 Mg m^−^^3^
*M**_r_* = 192.26	Mo *K*α radiation, λ = 0.71073 Å
Orthorhombic, *P**n**a*2_1_	Cell parameters from 1984 reflections
*a* = 6.9683 (7) Å	θ = 2.8–27.7°
*b* = 7.9683 (8) Å	µ = 0.08 mm^−^^1^
*c* = 18.975 (2) Å	*T* = 293 K
*V* = 1053.60 (19) Å^3^	Block, colourless
*Z* = 4	0.48 × 0.48 × 0.40 mm
*F*(000) = 416	

### N-(4-Methoxyphenyl)piperazine (I) Data collection

**Table:**

Oxford Diffraction Xcalibur with Sapphire CCD diffractometer	1984 independent reflections
Radiation source: Enhance (Mo) X-ray Source	1545 reflections with *I* > 2σ(*I*)
Graphite monochromator	*R*_int_ = 0.012
ω scans	θ_max_ = 27.7°, θ_min_ = 2.8°
Absorption correction: multi-scan (CrysAlis RED; Oxford Diffraction, 2009)	*h* = −8→8
*T*_min_ = 0.814, *T*_max_ = 0.969	*k* = −10→10
4066 measured reflections	*l* = −24→21

### N-(4-Methoxyphenyl)piperazine (I) Refinement

**Table:**

Refinement on *F*^2^	Primary atom site location: dual
Least-squares matrix: full	Hydrogen site location: mixed
*R*[*F*^2^ > 2σ(*F*^2^)] = 0.036	H atoms treated by a mixture of independent and constrained refinement
*wR*(*F*^2^) = 0.088	*w* = 1/[σ^2^(*F*_o_^2^) + (0.0466*P*)^2^ + 0.042*P*] where *P* = (*F*_o_^2^ + 2*F*_c_^2^)/3
*S* = 1.06	(Δ/σ)_max_ < 0.001
1984 reflections	Δρ_max_ = 0.10 e Å^−^^3^
131 parameters	Δρ_min_ = −0.12 e Å^−^^3^
1 restraint	Absolute structure: Flack x determined using 546 quotients [(*I*^+^)-(*I*^-^)]/[(*I*^+^)+(*I*^-^)] (Parsons *et al.*, 2013)

### N-(4-Methoxyphenyl)piperazine (I) Special details

**Table:**

Geometry. All esds (except the esd in the dihedral angle between two l.s. planes) are estimated using the full covariance matrix. The cell esds are taken into account individually in the estimation of esds in distances, angles and torsion angles; correlations between esds in cell parameters are only used when they are defined by crystal symmetry. An approximate (isotropic) treatment of cell esds is used for estimating esds involving l.s. planes.

### N-(4-Methoxyphenyl)piperazine (I) Fractional atomic coordinates and isotropic or equivalent isotropic displacement parameters (Å^2^)

**Table:**

	*x*	*y*	*z*	*U*_iso_*/*U*_eq_	
N1	0.7092 (4)	0.4549 (3)	0.62326 (14)	0.0784 (6)	
H1	0.790 (5)	0.480 (4)	0.6597 (19)	0.094*	
C2	0.8074 (4)	0.4800 (3)	0.55762 (17)	0.0709 (7)	
H2A	0.9239	0.4134	0.5568	0.085*	
H2B	0.8428	0.5972	0.5528	0.085*	
C3	0.6799 (3)	0.4296 (3)	0.49739 (15)	0.0617 (6)	
H3A	0.7453	0.4509	0.4532	0.074*	
H3B	0.6531	0.3103	0.5002	0.074*	
N4	0.4997 (3)	0.5231 (2)	0.49904 (11)	0.0502 (4)	
C5	0.4049 (3)	0.5099 (3)	0.56726 (13)	0.0582 (6)	
H5A	0.3622	0.3954	0.5746	0.070*	
H5B	0.2930	0.5823	0.5680	0.070*	
C6	0.5390 (4)	0.5596 (4)	0.62554 (15)	0.0716 (7)	
H6A	0.5749	0.6765	0.6203	0.086*	
H6B	0.4754	0.5464	0.6707	0.086*	
C21	0.3791 (3)	0.5080 (2)	0.43961 (12)	0.0465 (5)	
C22	0.4222 (3)	0.4034 (3)	0.38284 (13)	0.0568 (6)	
H22	0.5303	0.3351	0.3848	0.068*	
C23	0.3067 (4)	0.3998 (3)	0.32390 (13)	0.0614 (6)	
H23	0.3391	0.3300	0.2864	0.074*	
C24	0.1445 (4)	0.4975 (3)	0.31948 (13)	0.0568 (7)	
C25	0.0979 (3)	0.6002 (3)	0.37552 (14)	0.0567 (6)	
H25	−0.0117	0.6665	0.3736	0.068*	
C26	0.2136 (3)	0.6042 (2)	0.43401 (13)	0.0539 (6)	
H26	0.1799	0.6739	0.4713	0.065*	
O24	0.0395 (3)	0.4861 (2)	0.25830 (11)	0.0798 (6)	
C27	−0.1153 (4)	0.5990 (5)	0.2494 (2)	0.1065 (12)	
H27A	−0.1652	0.5889	0.2024	0.160*	
H27B	−0.0713	0.7118	0.2570	0.160*	
H27C	−0.2145	0.5731	0.2828	0.160*	

### N-(4-Methoxyphenyl)piperazine (I) Atomic displacement parameters (Å^2^)

**Table:**

	*U*^11^	*U*^22^	*U*^33^	*U*^12^	*U*^13^	*U*^23^
N1	0.0715 (15)	0.0896 (15)	0.0739 (15)	−0.0055 (13)	−0.0133 (12)	0.0146 (12)
C2	0.0555 (13)	0.0649 (14)	0.0923 (19)	0.0004 (12)	−0.0035 (15)	0.0092 (14)
C3	0.0523 (12)	0.0568 (12)	0.0761 (15)	0.0058 (11)	0.0081 (13)	0.0058 (12)
N4	0.0450 (8)	0.0497 (9)	0.0560 (11)	0.0007 (7)	0.0087 (9)	0.0028 (8)
C5	0.0531 (12)	0.0625 (13)	0.0588 (14)	−0.0064 (10)	0.0095 (12)	0.0008 (10)
C6	0.0707 (16)	0.0855 (18)	0.0586 (14)	−0.0130 (14)	0.0012 (13)	−0.0009 (12)
C21	0.0475 (11)	0.0390 (11)	0.0532 (13)	−0.0030 (9)	0.0137 (10)	0.0035 (10)
C22	0.0580 (14)	0.0531 (13)	0.0594 (15)	0.0095 (10)	0.0132 (13)	0.0007 (10)
C23	0.0710 (15)	0.0595 (14)	0.0538 (15)	0.0044 (13)	0.0187 (13)	−0.0080 (10)
C24	0.0574 (14)	0.0637 (15)	0.0492 (15)	−0.0055 (11)	0.0077 (12)	0.0029 (10)
C25	0.0487 (12)	0.0570 (13)	0.0643 (15)	0.0047 (10)	0.0089 (12)	−0.0001 (11)
C26	0.0535 (12)	0.0500 (12)	0.0581 (14)	0.0031 (10)	0.0123 (12)	−0.0069 (10)
O24	0.0771 (12)	0.1029 (15)	0.0595 (12)	0.0020 (11)	−0.0017 (11)	−0.0059 (10)
C27	0.077 (2)	0.154 (4)	0.089 (2)	0.0160 (18)	−0.0205 (19)	−0.007 (2)

### N-(4-Methoxyphenyl)piperazine (I) Geometric parameters (Å, º)

**Table:**

N1—C2	1.435 (4)	C21—C26	1.389 (3)
N1—C6	1.451 (4)	C21—C22	1.395 (3)
N1—H1	0.91 (4)	C22—C23	1.378 (4)
C2—C3	1.502 (4)	C22—H22	0.9300
C2—H2A	0.9700	C23—C24	1.375 (3)
C2—H2B	0.9700	C23—H23	0.9300
C3—N4	1.460 (3)	C24—O24	1.375 (3)
C3—H3A	0.9700	C24—C25	1.380 (4)
C3—H3B	0.9700	C25—C26	1.372 (4)
N4—C21	1.411 (3)	C25—H25	0.9300
N4—C5	1.457 (3)	C26—H26	0.9300
C5—C6	1.501 (4)	O24—C27	1.415 (4)
C5—H5A	0.9700	C27—H27A	0.9600
C5—H5B	0.9700	C27—H27B	0.9600
C6—H6A	0.9700	C27—H27C	0.9600
C6—H6B	0.9700		
			
C2—N1—C6	109.6 (2)	C5—C6—H6B	109.7
C2—N1—H1	110 (2)	H6A—C6—H6B	108.2
C6—N1—H1	111 (2)	C26—C21—C22	116.7 (2)
N1—C2—C3	109.9 (2)	C26—C21—N4	120.58 (19)
N1—C2—H2A	109.7	C22—C21—N4	122.66 (19)
C3—C2—H2A	109.7	C23—C22—C21	120.9 (2)
N1—C2—H2B	109.7	C23—C22—H22	119.6
C3—C2—H2B	109.7	C21—C22—H22	119.6
H2A—C2—H2B	108.2	C24—C23—C22	121.2 (2)
N4—C3—C2	110.8 (2)	C24—C23—H23	119.4
N4—C3—H3A	109.5	C22—C23—H23	119.4
C2—C3—H3A	109.5	O24—C24—C23	116.8 (2)
N4—C3—H3B	109.5	O24—C24—C25	124.4 (2)
C2—C3—H3B	109.5	C23—C24—C25	118.8 (2)
H3A—C3—H3B	108.1	C26—C25—C24	119.9 (2)
C21—N4—C5	115.72 (15)	C26—C25—H25	120.0
C21—N4—C3	116.8 (2)	C24—C25—H25	120.0
C5—N4—C3	111.8 (2)	C25—C26—C21	122.5 (2)
N4—C5—C6	110.71 (18)	C25—C26—H26	118.8
N4—C5—H5A	109.5	C21—C26—H26	118.8
C6—C5—H5A	109.5	C24—O24—C27	117.7 (2)
N4—C5—H5B	109.5	O24—C27—H27A	109.5
C6—C5—H5B	109.5	O24—C27—H27B	109.5
H5A—C5—H5B	108.1	H27A—C27—H27B	109.5
N1—C6—C5	109.6 (2)	O24—C27—H27C	109.5
N1—C6—H6A	109.7	H27A—C27—H27C	109.5
C5—C6—H6A	109.7	H27B—C27—H27C	109.5
N1—C6—H6B	109.7		
			
C6—N1—C2—C3	−61.6 (3)	C26—C21—C22—C23	1.3 (3)
N1—C2—C3—N4	57.0 (3)	N4—C21—C22—C23	−176.0 (2)
C2—C3—N4—C21	170.30 (19)	C21—C22—C23—C24	−0.8 (3)
C2—C3—N4—C5	−53.1 (3)	C22—C23—C24—O24	179.5 (2)
C21—N4—C5—C6	−169.38 (19)	C22—C23—C24—C25	−0.1 (3)
C3—N4—C5—C6	53.5 (2)	O24—C24—C25—C26	−179.1 (2)
C2—N1—C6—C5	61.8 (3)	C23—C24—C25—C26	0.5 (3)
N4—C5—C6—N1	−57.4 (3)	C24—C25—C26—C21	0.1 (3)
C5—N4—C21—C26	50.9 (2)	C22—C21—C26—C25	−1.0 (3)
C3—N4—C21—C26	−174.17 (19)	N4—C21—C26—C25	176.40 (19)
C5—N4—C21—C22	−131.9 (2)	C23—C24—O24—C27	−173.1 (3)
C3—N4—C21—C22	3.0 (3)	C25—C24—O24—C27	6.6 (4)

### N-(4-Methoxyphenyl)piperazine (I) Hydrogen-bond geometry (Å, º)

**Table:**

*D*—H···*A*	*D*—H	H···*A*	*D*···*A*	*D*—H···*A*
N1—H1···O24^i^	0.91 (4)	2.23 (4)	3.139 (3)	171 (3)

Symmetry code: (i) −*x*+1, −*y*+1, *z*+1/2.

### 4-(4-Methoxyphenyl)piperazin-1-ium 3,5-dinitrobenzoate (II) Crystal data

**Table:**

C_11_H_17_N_2_O^+^·C_7_H_3_N_2_O_6_^−^	*Z* = 2
*M**_r_* = 404.38	*F*(000) = 424
Triclinic, *P*1	*D*_x_ = 1.366 Mg m^−^^3^
*a* = 7.4365 (4) Å	Mo *K*α radiation, λ = 0.71073 Å
*b* = 10.6276 (6) Å	Cell parameters from 4210 reflections
*c* = 13.2700 (6) Å	θ = 2.7–27.8°
α = 92.238 (4)°	µ = 0.11 mm^−^^1^
β = 97.057 (4)°	*T* = 293 K
γ = 108.618 (5)°	Block, yellow
*V* = 982.92 (9) Å^3^	0.50 × 0.48 × 0.48 mm

### 4-(4-Methoxyphenyl)piperazin-1-ium 3,5-dinitrobenzoate (II) Data collection

**Table:**

Oxford Diffraction Xcalibur with Sapphire CCD diffractometer	4202 independent reflections
Radiation source: Enhance (Mo) X-ray Source	3057 reflections with *I* > 2σ(*I*)
Graphite monochromator	*R*_int_ = 0.011
ω scans	θ_max_ = 27.5°, θ_min_ = 2.7°
Absorption correction: multi-scan (CrysAlis RED; Oxford Diffraction, 2009)	*h* = −7→9
*T*_min_ = 0.765, *T*_max_ = 0.950	*k* = −13→13
7013 measured reflections	*l* = −16→9

### 4-(4-Methoxyphenyl)piperazin-1-ium 3,5-dinitrobenzoate (II) Refinement

**Table:**

Refinement on *F*^2^	Primary atom site location: dual
Least-squares matrix: full	Hydrogen site location: mixed
*R*[*F*^2^ > 2σ(*F*^2^)] = 0.041	H atoms treated by a mixture of independent and constrained refinement
*wR*(*F*^2^) = 0.110	*w* = 1/[σ^2^(*F*_o_^2^) + (0.0482*P*)^2^ + 0.2082*P*] where *P* = (*F*_o_^2^ + 2*F*_c_^2^)/3
*S* = 1.02	(Δ/σ)_max_ < 0.001
4202 reflections	Δρ_max_ = 0.15 e Å^−^^3^
268 parameters	Δρ_min_ = −0.19 e Å^−^^3^
0 restraints	

### 4-(4-Methoxyphenyl)piperazin-1-ium 3,5-dinitrobenzoate (II) Special details

**Table:**

Geometry. All esds (except the esd in the dihedral angle between two l.s. planes) are estimated using the full covariance matrix. The cell esds are taken into account individually in the estimation of esds in distances, angles and torsion angles; correlations between esds in cell parameters are only used when they are defined by crystal symmetry. An approximate (isotropic) treatment of cell esds is used for estimating esds involving l.s. planes.

### 4-(4-Methoxyphenyl)piperazin-1-ium 3,5-dinitrobenzoate (II) Fractional atomic coordinates and isotropic or equivalent isotropic displacement parameters (Å^2^)

**Table:**

	*x*	*y*	*z*	*U*_iso_*/*U*_eq_	
N1	0.4909 (2)	0.44996 (14)	0.34081 (10)	0.0521 (3)	
H11	0.578 (3)	0.5249 (19)	0.3837 (14)	0.063*	
H12	0.464 (3)	0.3711 (18)	0.3776 (14)	0.063*	
C2	0.3094 (3)	0.47725 (16)	0.31197 (13)	0.0579 (4)	
H2A	0.2620	0.4993	0.3729	0.070*	
H2B	0.2140	0.3982	0.2761	0.070*	
C3	0.3399 (3)	0.59140 (16)	0.24455 (12)	0.0518 (4)	
H3A	0.2191	0.6068	0.2249	0.062*	
H3B	0.4290	0.6719	0.2819	0.062*	
N4	0.41580 (19)	0.56126 (12)	0.15311 (9)	0.0475 (3)	
C5	0.5974 (3)	0.54000 (17)	0.18174 (12)	0.0532 (4)	
H5A	0.6890	0.6204	0.2180	0.064*	
H5B	0.6473	0.5205	0.1209	0.064*	
C6	0.5732 (3)	0.42574 (17)	0.24871 (13)	0.0601 (5)	
H6A	0.4893	0.3441	0.2105	0.072*	
H6B	0.6967	0.4147	0.2689	0.072*	
C21	0.4128 (2)	0.65236 (14)	0.07691 (11)	0.0443 (3)	
C22	0.2377 (2)	0.65974 (17)	0.03241 (12)	0.0555 (4)	
H22	0.1256	0.6063	0.0536	0.067*	
C23	0.2264 (2)	0.74413 (18)	−0.04220 (13)	0.0572 (4)	
H23	0.1074	0.7474	−0.0706	0.069*	
C24	0.3915 (2)	0.82446 (16)	−0.07540 (11)	0.0468 (4)	
C25	0.5662 (2)	0.81844 (16)	−0.03278 (11)	0.0477 (4)	
H25	0.6779	0.8715	−0.0546	0.057*	
C26	0.5764 (2)	0.73326 (16)	0.04294 (11)	0.0477 (4)	
H26	0.6957	0.7305	0.0714	0.057*	
O24	0.36537 (16)	0.90394 (12)	−0.15109 (9)	0.0609 (3)	
C27	0.5312 (2)	0.98606 (18)	−0.18771 (14)	0.0595 (4)	
H27A	0.4941	1.0364	−0.2401	0.089*	
H27B	0.6143	1.0459	−0.1327	0.089*	
H27C	0.5973	0.9314	−0.2151	0.089*	
C31	0.75477 (19)	0.90885 (13)	0.44906 (10)	0.0370 (3)	
C32	0.74671 (19)	1.02627 (13)	0.49332 (11)	0.0379 (3)	
H32	0.6919	1.0266	0.5526	0.045*	
C33	0.8211 (2)	1.14339 (13)	0.44845 (11)	0.0397 (3)	
C34	0.9027 (2)	1.14837 (15)	0.36080 (11)	0.0425 (3)	
H34	0.9502	1.2278	0.3310	0.051*	
C35	0.91080 (19)	1.03023 (15)	0.31926 (10)	0.0416 (3)	
C36	0.8395 (2)	0.91119 (14)	0.36128 (11)	0.0413 (3)	
H36	0.8480	0.8331	0.3312	0.050*	
C37	0.6792 (2)	0.77976 (14)	0.49869 (12)	0.0442 (3)	
O31	0.70674 (18)	0.67975 (10)	0.46073 (10)	0.0624 (3)	
O32	0.59993 (18)	0.78544 (11)	0.57568 (10)	0.0617 (3)	
N33	0.8205 (2)	1.26892 (13)	0.49865 (12)	0.0556 (4)	
O33	0.7947 (3)	1.26858 (14)	0.58726 (12)	0.0877 (5)	
O34	0.8460 (2)	1.36548 (11)	0.44914 (12)	0.0745 (4)	
N35	1.00278 (19)	1.03268 (17)	0.22712 (11)	0.0575 (4)	
O35	0.9969 (2)	0.92646 (16)	0.18661 (11)	0.0886 (5)	
O36	1.08186 (19)	1.14064 (15)	0.19653 (10)	0.0723 (4)	

### 4-(4-Methoxyphenyl)piperazin-1-ium 3,5-dinitrobenzoate (II) Atomic displacement parameters (Å^2^)

**Table:**

	*U*^11^	*U*^22^	*U*^33^	*U*^12^	*U*^13^	*U*^23^
N1	0.0813 (10)	0.0340 (7)	0.0413 (7)	0.0163 (7)	0.0151 (7)	0.0085 (6)
C2	0.0783 (12)	0.0465 (9)	0.0517 (9)	0.0171 (8)	0.0255 (9)	0.0128 (7)
C3	0.0688 (11)	0.0490 (9)	0.0434 (8)	0.0220 (8)	0.0200 (8)	0.0106 (7)
N4	0.0625 (8)	0.0452 (7)	0.0365 (6)	0.0178 (6)	0.0121 (6)	0.0052 (5)
C5	0.0726 (11)	0.0562 (9)	0.0390 (8)	0.0285 (8)	0.0170 (8)	0.0091 (7)
C6	0.0932 (14)	0.0516 (9)	0.0463 (9)	0.0353 (9)	0.0179 (9)	0.0066 (7)
C21	0.0539 (9)	0.0432 (8)	0.0331 (7)	0.0126 (7)	0.0060 (6)	0.0004 (6)
C22	0.0475 (9)	0.0609 (10)	0.0494 (9)	0.0045 (8)	0.0083 (7)	0.0100 (8)
C23	0.0430 (9)	0.0721 (11)	0.0525 (9)	0.0141 (8)	0.0020 (7)	0.0152 (8)
C24	0.0511 (9)	0.0534 (9)	0.0352 (7)	0.0161 (7)	0.0056 (6)	0.0062 (7)
C25	0.0458 (9)	0.0581 (9)	0.0386 (8)	0.0140 (7)	0.0101 (6)	0.0090 (7)
C26	0.0475 (9)	0.0591 (9)	0.0378 (8)	0.0186 (7)	0.0067 (6)	0.0069 (7)
O24	0.0505 (7)	0.0792 (8)	0.0544 (7)	0.0201 (6)	0.0080 (5)	0.0293 (6)
C27	0.0565 (10)	0.0651 (11)	0.0575 (10)	0.0177 (8)	0.0105 (8)	0.0234 (9)
C31	0.0330 (7)	0.0355 (7)	0.0397 (7)	0.0091 (5)	−0.0009 (5)	0.0061 (6)
C32	0.0348 (7)	0.0413 (7)	0.0382 (7)	0.0127 (6)	0.0052 (6)	0.0074 (6)
C33	0.0377 (7)	0.0351 (7)	0.0466 (8)	0.0131 (6)	0.0030 (6)	0.0062 (6)
C34	0.0367 (8)	0.0421 (8)	0.0464 (8)	0.0092 (6)	0.0036 (6)	0.0155 (6)
C35	0.0328 (7)	0.0539 (9)	0.0357 (7)	0.0107 (6)	0.0038 (6)	0.0073 (6)
C36	0.0385 (8)	0.0398 (7)	0.0433 (8)	0.0120 (6)	0.0008 (6)	−0.0011 (6)
C37	0.0415 (8)	0.0353 (7)	0.0505 (9)	0.0077 (6)	−0.0015 (7)	0.0091 (6)
O31	0.0784 (8)	0.0349 (6)	0.0698 (8)	0.0157 (5)	0.0036 (6)	0.0028 (5)
O32	0.0726 (8)	0.0484 (6)	0.0700 (8)	0.0186 (6)	0.0285 (7)	0.0260 (6)
N33	0.0577 (9)	0.0405 (7)	0.0724 (10)	0.0205 (6)	0.0109 (7)	0.0057 (7)
O33	0.1379 (14)	0.0651 (9)	0.0746 (10)	0.0460 (9)	0.0368 (9)	−0.0020 (7)
O34	0.0822 (9)	0.0374 (6)	0.1060 (11)	0.0211 (6)	0.0133 (8)	0.0183 (7)
N35	0.0442 (8)	0.0778 (11)	0.0466 (8)	0.0131 (7)	0.0107 (6)	0.0044 (8)
O35	0.0877 (11)	0.0931 (11)	0.0767 (9)	0.0117 (8)	0.0374 (8)	−0.0187 (8)
O36	0.0611 (8)	0.0951 (10)	0.0654 (8)	0.0219 (7)	0.0285 (6)	0.0330 (7)

### 4-(4-Methoxyphenyl)piperazin-1-ium 3,5-dinitrobenzoate (II) Geometric parameters (Å, º)

**Table:**

N1—C2	1.478 (2)	C25—C26	1.390 (2)
N1—C6	1.481 (2)	C25—H25	0.9300
N1—H11	0.96 (2)	C26—H26	0.9300
N1—H12	0.965 (18)	O24—C27	1.4181 (19)
C2—C3	1.511 (2)	C27—H27A	0.9600
C2—H2A	0.9700	C27—H27B	0.9600
C2—H2B	0.9700	C27—H27C	0.9600
C3—N4	1.4654 (19)	C31—C32	1.3803 (19)
C3—H3A	0.9700	C31—C36	1.388 (2)
C3—H3B	0.9700	C31—C37	1.5169 (19)
N4—C21	1.4301 (18)	C32—C33	1.3817 (19)
N4—C5	1.448 (2)	C32—H32	0.9300
C5—C6	1.510 (2)	C33—C34	1.372 (2)
C5—H5A	0.9700	C33—N33	1.4693 (19)
C5—H5B	0.9700	C34—C35	1.374 (2)
C6—H6A	0.9700	C34—H34	0.9300
C6—H6B	0.9700	C35—C36	1.376 (2)
C21—C26	1.386 (2)	C35—N35	1.4696 (19)
C21—C22	1.389 (2)	C36—H36	0.9300
C22—C23	1.374 (2)	C37—O31	1.2440 (18)
C22—H22	0.9300	C37—O32	1.2495 (19)
C23—C24	1.387 (2)	N33—O33	1.2143 (19)
C23—H23	0.9300	N33—O34	1.2165 (17)
C24—O24	1.3724 (18)	N35—O35	1.2169 (19)
C24—C25	1.373 (2)	N35—O36	1.2211 (18)
			
C2—N1—C6	110.43 (13)	O24—C24—C23	116.04 (14)
C2—N1—H11	108.2 (11)	C25—C24—C23	119.13 (14)
C6—N1—H11	111.1 (11)	C24—C25—C26	120.16 (14)
C2—N1—H12	108.4 (11)	C24—C25—H25	119.9
C6—N1—H12	108.7 (10)	C26—C25—H25	119.9
H11—N1—H12	110.0 (15)	C21—C26—C25	121.40 (15)
N1—C2—C3	110.47 (14)	C21—C26—H26	119.3
N1—C2—H2A	109.6	C25—C26—H26	119.3
C3—C2—H2A	109.6	C24—O24—C27	117.47 (13)
N1—C2—H2B	109.6	O24—C27—H27A	109.5
C3—C2—H2B	109.6	O24—C27—H27B	109.5
H2A—C2—H2B	108.1	H27A—C27—H27B	109.5
N4—C3—C2	110.40 (13)	O24—C27—H27C	109.5
N4—C3—H3A	109.6	H27A—C27—H27C	109.5
C2—C3—H3A	109.6	H27B—C27—H27C	109.5
N4—C3—H3B	109.6	C32—C31—C36	119.36 (12)
C2—C3—H3B	109.6	C32—C31—C37	120.07 (13)
H3A—C3—H3B	108.1	C36—C31—C37	120.52 (13)
C21—N4—C5	115.97 (12)	C31—C32—C33	119.18 (13)
C21—N4—C3	113.26 (12)	C31—C32—H32	120.4
C5—N4—C3	109.90 (12)	C33—C32—H32	120.4
N4—C5—C6	110.50 (14)	C34—C33—C32	122.74 (13)
N4—C5—H5A	109.6	C34—C33—N33	118.33 (13)
C6—C5—H5A	109.6	C32—C33—N33	118.88 (13)
N4—C5—H5B	109.6	C33—C34—C35	116.73 (13)
C6—C5—H5B	109.6	C33—C34—H34	121.6
H5A—C5—H5B	108.1	C35—C34—H34	121.6
N1—C6—C5	111.08 (13)	C34—C35—C36	122.71 (13)
N1—C6—H6A	109.4	C34—C35—N35	118.15 (13)
C5—C6—H6A	109.4	C36—C35—N35	119.13 (14)
N1—C6—H6B	109.4	C35—C36—C31	119.26 (13)
C5—C6—H6B	109.4	C35—C36—H36	120.4
H6A—C6—H6B	108.0	C31—C36—H36	120.4
C26—C21—C22	117.42 (14)	O31—C37—O32	126.39 (14)
C26—C21—N4	123.45 (14)	O31—C37—C31	117.10 (14)
C22—C21—N4	119.12 (14)	O32—C37—C31	116.49 (13)
C23—C22—C21	121.53 (15)	O33—N33—O34	124.64 (15)
C23—C22—H22	119.2	O33—N33—C33	117.22 (13)
C21—C22—H22	119.2	O34—N33—C33	118.14 (15)
C22—C23—C24	120.35 (15)	O35—N35—O36	124.03 (15)
C22—C23—H23	119.8	O35—N35—C35	117.67 (15)
C24—C23—H23	119.8	O36—N35—C35	118.29 (15)
O24—C24—C25	124.83 (14)		
			
C6—N1—C2—C3	−55.07 (17)	C36—C31—C32—C33	0.8 (2)
N1—C2—C3—N4	57.89 (18)	C37—C31—C32—C33	178.20 (12)
C2—C3—N4—C21	168.46 (14)	C31—C32—C33—C34	0.3 (2)
C2—C3—N4—C5	−60.05 (18)	C31—C32—C33—N33	−177.08 (13)
C21—N4—C5—C6	−170.46 (12)	C32—C33—C34—C35	−1.1 (2)
C3—N4—C5—C6	59.49 (16)	N33—C33—C34—C35	176.30 (13)
C2—N1—C6—C5	54.81 (19)	C33—C34—C35—C36	0.8 (2)
N4—C5—C6—N1	−57.29 (19)	C33—C34—C35—N35	−178.10 (13)
C5—N4—C21—C26	−10.3 (2)	C34—C35—C36—C31	0.3 (2)
C3—N4—C21—C26	118.13 (17)	N35—C35—C36—C31	179.18 (12)
C5—N4—C21—C22	168.48 (14)	C32—C31—C36—C35	−1.1 (2)
C3—N4—C21—C22	−63.10 (18)	C37—C31—C36—C35	−178.48 (12)
C26—C21—C22—C23	−0.2 (2)	C32—C31—C37—O31	−173.38 (14)
N4—C21—C22—C23	−179.05 (15)	C36—C31—C37—O31	4.0 (2)
C21—C22—C23—C24	0.2 (3)	C32—C31—C37—O32	4.7 (2)
C22—C23—C24—O24	179.14 (16)	C36—C31—C37—O32	−177.93 (13)
C22—C23—C24—C25	0.0 (3)	C34—C33—N33—O33	−161.49 (16)
O24—C24—C25—C26	−179.33 (14)	C32—C33—N33—O33	16.0 (2)
C23—C24—C25—C26	−0.3 (2)	C34—C33—N33—O34	18.4 (2)
C22—C21—C26—C25	−0.1 (2)	C32—C33—N33—O34	−164.13 (14)
N4—C21—C26—C25	178.71 (14)	C34—C35—N35—O35	−174.75 (15)
C24—C25—C26—C21	0.3 (2)	C36—C35—N35—O35	6.3 (2)
C25—C24—O24—C27	−0.2 (2)	C34—C35—N35—O36	6.0 (2)
C23—C24—O24—C27	−179.20 (15)	C36—C35—N35—O36	−172.92 (14)

### 4-(4-Methoxyphenyl)piperazin-1-ium 3,5-dinitrobenzoate (II) Hydrogen-bond geometry (Å, º)

**Table:**

*D*—H···*A*	*D*—H	H···*A*	*D*···*A*	*D*—H···*A*
N1—H11···O31	0.96 (2)	1.814 (19)	2.7638 (18)	169 (2)
N1—H12···O32^i^	0.964 (18)	1.740 (18)	2.6953 (18)	170.5 (17)

Symmetry code: (i) −*x*+1, −*y*+1, −*z*+1.

### 4-(4-Methoxyphenyl)piperazin-1-ium 2,4,6-trinitrophenolate (III) Crystal data

**Table:**

C_11_H_17_N_2_O^+^·C_6_H_2_N_3_O_7_^−^	*F*(000) = 880
*M**_r_* = 421.37	*D*_x_ = 1.428 Mg m^−^^3^
Monoclinic, *P*2_1_/*n*	Mo *K*α radiation, λ = 0.71073 Å
*a* = 8.7568 (6) Å	Cell parameters from 4353 reflections
*b* = 6.6292 (5) Å	θ = 2.8–27.9°
*c* = 34.024 (2) Å	µ = 0.12 mm^−^^1^
β = 96.987 (6)°	*T* = 293 K
*V* = 1960.4 (2) Å^3^	Plate, yellow
*Z* = 4	0.48 × 0.42 × 0.20 mm

### 4-(4-Methoxyphenyl)piperazin-1-ium 2,4,6-trinitrophenolate (III) Data collection

**Table:**

Oxford Diffraction Xcalibur with Sapphire CCD diffractometer	4353 independent reflections
Radiation source: Enhance (Mo) X-ray Source	2844 reflections with *I* > 2σ(*I*)
Graphite monochromator	*R*_int_ = 0.023
ω scans	θ_max_ = 27.9°, θ_min_ = 2.8°
Absorption correction: multi-scan (CrysAlis RED; Oxford Diffraction, 2009)	*h* = −11→11
*T*_min_ = 0.844, *T*_max_ = 0.977	*k* = −8→8
14483 measured reflections	*l* = −40→42

### 4-(4-Methoxyphenyl)piperazin-1-ium 2,4,6-trinitrophenolate (III) Refinement

**Table:**

Refinement on *F*^2^	Primary atom site location: dual
Least-squares matrix: full	Hydrogen site location: mixed
*R*[*F*^2^ > 2σ(*F*^2^)] = 0.058	H atoms treated by a mixture of independent and constrained refinement
*wR*(*F*^2^) = 0.136	*w* = 1/[σ^2^(*F*_o_^2^) + (0.0408*P*)^2^ + 1.052*P*] where *P* = (*F*_o_^2^ + 2*F*_c_^2^)/3
*S* = 1.09	(Δ/σ)_max_ = 0.001
4353 reflections	Δρ_max_ = 0.18 e Å^−^^3^
333 parameters	Δρ_min_ = −0.18 e Å^−^^3^
216 restraints	

### 4-(4-Methoxyphenyl)piperazin-1-ium 2,4,6-trinitrophenolate (III) Special details

**Table:**

Geometry. All esds (except the esd in the dihedral angle between two l.s. planes) are estimated using the full covariance matrix. The cell esds are taken into account individually in the estimation of esds in distances, angles and torsion angles; correlations between esds in cell parameters are only used when they are defined by crystal symmetry. An approximate (isotropic) treatment of cell esds is used for estimating esds involving l.s. planes.

### 4-(4-Methoxyphenyl)piperazin-1-ium 2,4,6-trinitrophenolate (III) Fractional atomic coordinates and isotropic or equivalent isotropic displacement parameters (Å^2^)

**Table:**

	*x*	*y*	*z*	*U*_iso_*/*U*_eq_	Occ. (<1)
N1	0.0738 (2)	0.1834 (3)	0.59884 (5)	0.0479 (5)	
H11	0.151 (3)	0.169 (4)	0.5830 (7)	0.058*	
H12	−0.019 (3)	0.169 (4)	0.5837 (7)	0.058*	
C2	0.0862 (3)	0.0294 (4)	0.63061 (6)	0.0525 (6)	
H2A	0.0870	−0.1042	0.6190	0.063*	
H2B	−0.0021	0.0387	0.6452	0.063*	
C3	0.2321 (3)	0.0615 (4)	0.65845 (7)	0.0500 (6)	
H3A	0.2374	−0.0366	0.6797	0.060*	
H3B	0.3205	0.0412	0.6443	0.060*	
N4	0.23682 (19)	0.2647 (3)	0.67506 (5)	0.0400 (4)	
C5	0.2311 (3)	0.4129 (4)	0.64346 (6)	0.0466 (6)	
H5A	0.3190	0.3950	0.6290	0.056*	
H5B	0.2362	0.5476	0.6547	0.056*	
C6	0.0844 (3)	0.3897 (4)	0.61558 (7)	0.0541 (6)	
H6A	−0.0033	0.4153	0.6297	0.065*	
H6B	0.0827	0.4874	0.5943	0.065*	
C21	0.3552 (2)	0.2940 (3)	0.70738 (6)	0.0395 (5)	
C22	0.3572 (3)	0.1707 (4)	0.74060 (6)	0.0484 (6)	
H22	0.2840	0.0692	0.7409	0.058*	
C23	0.4660 (3)	0.1966 (4)	0.77304 (6)	0.0544 (7)	
H23	0.4667	0.1108	0.7947	0.065*	
C24	0.5738 (3)	0.3482 (4)	0.77367 (6)	0.0528 (6)	
C25	0.5737 (3)	0.4715 (4)	0.74137 (7)	0.0562 (7)	
H25	0.6457	0.5747	0.7415	0.067*	
C26	0.4657 (3)	0.4422 (4)	0.70826 (6)	0.0487 (6)	
H26	0.4684	0.5247	0.6862	0.058*	
O24	0.6749 (2)	0.3618 (3)	0.80799 (5)	0.0735 (6)	
C27	0.7871 (4)	0.5171 (6)	0.81017 (8)	0.1008 (13)	
H27A	0.8500	0.5107	0.8353	0.151*	
H27B	0.8505	0.4998	0.7893	0.151*	
H27C	0.7368	0.6458	0.8074	0.151*	
C31	0.3734 (2)	0.2329 (3)	0.52703 (6)	0.0356 (5)	
O31	0.32183 (18)	0.2105 (3)	0.55895 (4)	0.0597 (5)	
C33	0.5987 (2)	0.2562 (3)	0.48889 (6)	0.0370 (5)	
H33	0.7047	0.2625	0.4888	0.044*	
C34	0.5007 (2)	0.2576 (3)	0.45381 (6)	0.0383 (5)	
C35	0.3436 (2)	0.2518 (3)	0.45352 (6)	0.0393 (5)	
H35	0.2796	0.2548	0.4296	0.047*	
C32	0.5365 (2)	0.2455 (3)	0.52377 (6)	0.0339 (4)	0.531 (16)
N32	0.6471 (5)	0.244 (2)	0.55966 (13)	0.036 (3)	0.531 (16)
O32	0.6107 (6)	0.1681 (18)	0.58975 (13)	0.068 (2)	0.531 (16)
O33	0.7762 (6)	0.3080 (16)	0.55749 (18)	0.0608 (18)	0.531 (16)
C42	0.5365 (2)	0.2455 (3)	0.52377 (6)	0.0339 (4)	0.469 (16)
N42	0.6434 (7)	0.242 (3)	0.55983 (17)	0.056 (5)	0.469 (16)
O42	0.6039 (6)	0.306 (2)	0.59065 (15)	0.071 (3)	0.469 (16)
O43	0.7755 (7)	0.191 (2)	0.5573 (2)	0.073 (2)	0.469 (16)
N34	0.5657 (2)	0.2627 (3)	0.41672 (5)	0.0514 (5)	
O34	0.7052 (2)	0.2600 (3)	0.41785 (5)	0.0689 (5)	
O35	0.4782 (2)	0.2684 (3)	0.38591 (5)	0.0800 (6)	
C36	0.2819 (2)	0.2416 (3)	0.48841 (6)	0.0370 (5)	0.62 (6)
N36	0.1151 (5)	0.2315 (18)	0.4864 (3)	0.056 (3)	0.62 (6)
O36	0.0431 (11)	0.178 (4)	0.4554 (4)	0.096 (4)	0.62 (6)
O37	0.0549 (12)	0.301 (3)	0.5138 (3)	0.084 (3)	0.62 (6)
C46	0.2819 (2)	0.2416 (3)	0.48841 (6)	0.0370 (5)	0.38 (6)
N46	0.1144 (7)	0.238 (3)	0.4844 (3)	0.055 (5)	0.38 (6)
O46	0.0441 (16)	0.215 (5)	0.4518 (4)	0.080 (5)	0.38 (6)
O47	0.0510 (15)	0.227 (9)	0.5144 (4)	0.089 (7)	0.38 (6)

### 4-(4-Methoxyphenyl)piperazin-1-ium 2,4,6-trinitrophenolate (III) Atomic displacement parameters (Å^2^)

**Table:**

	*U*^11^	*U*^22^	*U*^33^	*U*^12^	*U*^13^	*U*^23^
N1	0.0325 (10)	0.0750 (15)	0.0359 (10)	−0.0069 (10)	0.0025 (8)	−0.0021 (10)
C2	0.0542 (14)	0.0582 (16)	0.0448 (13)	−0.0104 (12)	0.0050 (11)	−0.0058 (11)
C3	0.0576 (14)	0.0484 (15)	0.0423 (12)	−0.0008 (11)	−0.0007 (10)	−0.0018 (11)
N4	0.0431 (10)	0.0433 (11)	0.0332 (8)	−0.0024 (8)	0.0034 (7)	0.0004 (8)
C5	0.0506 (13)	0.0499 (14)	0.0379 (11)	−0.0036 (11)	0.0001 (10)	0.0040 (10)
C6	0.0508 (14)	0.0640 (17)	0.0457 (13)	0.0036 (12)	−0.0017 (11)	0.0073 (12)
C21	0.0395 (11)	0.0485 (14)	0.0314 (10)	−0.0006 (10)	0.0080 (8)	−0.0022 (9)
C22	0.0515 (13)	0.0573 (15)	0.0371 (12)	−0.0095 (12)	0.0078 (10)	0.0040 (10)
C23	0.0574 (15)	0.0734 (18)	0.0328 (11)	−0.0057 (13)	0.0074 (10)	0.0102 (11)
C24	0.0456 (13)	0.0809 (19)	0.0312 (11)	−0.0042 (13)	0.0024 (9)	−0.0026 (12)
C25	0.0540 (14)	0.0710 (18)	0.0425 (13)	−0.0193 (13)	0.0018 (11)	0.0005 (12)
C26	0.0535 (14)	0.0582 (16)	0.0341 (11)	−0.0094 (12)	0.0039 (10)	0.0061 (10)
O24	0.0634 (11)	0.1146 (17)	0.0390 (9)	−0.0199 (11)	−0.0082 (8)	0.0053 (10)
C27	0.082 (2)	0.160 (4)	0.0538 (17)	−0.051 (2)	−0.0169 (15)	0.003 (2)
C31	0.0367 (10)	0.0329 (11)	0.0372 (11)	0.0006 (9)	0.0038 (8)	−0.0029 (9)
O31	0.0439 (9)	0.0975 (15)	0.0388 (8)	−0.0007 (9)	0.0093 (7)	0.0006 (9)
C33	0.0345 (10)	0.0303 (11)	0.0469 (11)	−0.0010 (9)	0.0081 (9)	0.0000 (9)
C34	0.0459 (12)	0.0340 (11)	0.0361 (10)	−0.0020 (10)	0.0089 (9)	0.0014 (9)
C35	0.0472 (12)	0.0343 (11)	0.0345 (10)	−0.0022 (10)	−0.0033 (9)	0.0010 (9)
C32	0.0349 (10)	0.0298 (11)	0.0351 (10)	−0.0001 (9)	−0.0032 (8)	−0.0027 (9)
N32	0.029 (4)	0.035 (5)	0.038 (5)	−0.004 (4)	−0.022 (4)	0.005 (4)
O32	0.072 (3)	0.094 (6)	0.034 (2)	−0.018 (3)	−0.0095 (17)	0.013 (3)
O33	0.033 (2)	0.075 (4)	0.070 (3)	−0.002 (3)	−0.0091 (17)	0.010 (3)
C42	0.0349 (10)	0.0298 (11)	0.0351 (10)	−0.0001 (9)	−0.0032 (8)	−0.0027 (9)
N42	0.062 (7)	0.050 (8)	0.058 (7)	0.003 (6)	0.022 (5)	−0.013 (6)
O42	0.069 (3)	0.095 (7)	0.044 (3)	0.005 (3)	−0.0066 (19)	−0.007 (3)
O43	0.036 (3)	0.096 (7)	0.080 (3)	0.014 (3)	−0.015 (2)	−0.013 (4)
N34	0.0644 (13)	0.0488 (12)	0.0427 (11)	−0.0041 (11)	0.0135 (10)	0.0020 (9)
O34	0.0609 (11)	0.0862 (14)	0.0653 (11)	0.0002 (11)	0.0300 (9)	0.0074 (10)
O35	0.0879 (14)	0.1139 (18)	0.0383 (9)	−0.0126 (13)	0.0081 (9)	0.0024 (11)
C36	0.0324 (10)	0.0363 (11)	0.0414 (11)	−0.0010 (9)	0.0011 (8)	−0.0007 (9)
N36	0.034 (4)	0.069 (5)	0.060 (6)	0.012 (4)	−0.016 (4)	−0.003 (4)
O36	0.049 (4)	0.147 (9)	0.085 (6)	−0.019 (4)	−0.020 (4)	−0.037 (7)
O37	0.046 (4)	0.129 (8)	0.079 (5)	0.024 (4)	0.018 (3)	0.006 (3)
C46	0.0324 (10)	0.0363 (11)	0.0414 (11)	−0.0010 (9)	0.0011 (8)	−0.0007 (9)
N46	0.045 (8)	0.075 (9)	0.049 (8)	−0.023 (7)	0.020 (7)	0.014 (7)
O46	0.043 (6)	0.138 (11)	0.054 (6)	0.015 (7)	−0.010 (5)	0.013 (8)
O47	0.038 (5)	0.18 (2)	0.043 (6)	−0.017 (7)	0.006 (4)	0.012 (6)

### 4-(4-Methoxyphenyl)piperazin-1-ium 2,4,6-trinitrophenolate (III) Geometric parameters (Å, º)

**Table:**

N1—C6	1.480 (3)	C26—H26	0.9300
N1—C2	1.481 (3)	O24—C27	1.419 (4)
N1—H11	0.92 (3)	C27—H27A	0.9600
N1—H12	0.91 (3)	C27—H27B	0.9600
C2—C3	1.510 (3)	C27—H27C	0.9600
C2—H2A	0.9700	C31—O31	1.235 (2)
C2—H2B	0.9700	C31—C32	1.448 (3)
C3—N4	1.460 (3)	C31—C36	1.455 (3)
C3—H3A	0.9700	C33—C32	1.366 (3)
C3—H3B	0.9700	C33—C34	1.383 (3)
N4—C21	1.430 (3)	C33—H33	0.9300
N4—C5	1.453 (3)	C34—C35	1.375 (3)
C5—C6	1.509 (3)	C34—N34	1.446 (3)
C5—H5A	0.9700	C35—C36	1.364 (3)
C5—H5B	0.9700	C35—H35	0.9300
C6—H6A	0.9700	C32—N32	1.463 (4)
C6—H6B	0.9700	N32—O32	1.218 (7)
C21—C26	1.377 (3)	N32—O33	1.218 (7)
C21—C22	1.393 (3)	N42—O43	1.218 (7)
C22—C23	1.378 (3)	N42—O42	1.222 (8)
C22—H22	0.9300	N34—O34	1.217 (2)
C23—C24	1.377 (3)	N34—O35	1.221 (2)
C23—H23	0.9300	C36—N36	1.455 (5)
C24—C25	1.369 (3)	N36—O36	1.213 (5)
C24—O24	1.380 (3)	N36—O37	1.218 (7)
C25—C26	1.393 (3)	N46—O46	1.213 (7)
C25—H25	0.9300	N46—O47	1.220 (8)
			
C6—N1—C2	111.14 (18)	C25—C24—O24	125.3 (2)
C6—N1—H11	107.7 (16)	C23—C24—O24	115.4 (2)
C2—N1—H11	111.3 (15)	C24—C25—C26	120.0 (2)
C6—N1—H12	108.7 (16)	C24—C25—H25	120.0
C2—N1—H12	108.8 (16)	C26—C25—H25	120.0
H11—N1—H12	109 (2)	C21—C26—C25	121.5 (2)
N1—C2—C3	110.02 (19)	C21—C26—H26	119.2
N1—C2—H2A	109.7	C25—C26—H26	119.2
C3—C2—H2A	109.7	C24—O24—C27	117.4 (2)
N1—C2—H2B	109.7	O24—C27—H27A	109.5
C3—C2—H2B	109.7	O24—C27—H27B	109.5
H2A—C2—H2B	108.2	H27A—C27—H27B	109.5
N4—C3—C2	110.8 (2)	O24—C27—H27C	109.5
N4—C3—H3A	109.5	H27A—C27—H27C	109.5
C2—C3—H3A	109.5	H27B—C27—H27C	109.5
N4—C3—H3B	109.5	O31—C31—C32	122.96 (18)
C2—C3—H3B	109.5	O31—C31—C36	125.38 (18)
H3A—C3—H3B	108.1	C32—C33—C34	118.62 (18)
C21—N4—C5	115.72 (17)	C32—C33—H33	120.7
C21—N4—C3	114.01 (17)	C34—C33—H33	120.7
C5—N4—C3	109.92 (17)	C35—C34—C33	121.41 (18)
N4—C5—C6	110.26 (19)	C35—C34—N34	119.60 (18)
N4—C5—H5A	109.6	C33—C34—N34	118.99 (19)
C6—C5—H5A	109.6	C36—C35—C34	119.79 (18)
N4—C5—H5B	109.6	C36—C35—H35	120.1
C6—C5—H5B	109.6	C34—C35—H35	120.1
H5A—C5—H5B	108.1	C33—C32—C31	124.80 (17)
N1—C6—C5	110.2 (2)	C33—C32—N32	115.6 (3)
N1—C6—H6A	109.6	C31—C32—N32	119.6 (3)
C5—C6—H6A	109.6	O32—N32—O33	122.3 (4)
N1—C6—H6B	109.6	O32—N32—C32	119.3 (6)
C5—C6—H6B	109.6	O33—N32—C32	118.2 (5)
H6A—C6—H6B	108.1	O43—N42—O42	122.0 (6)
C26—C21—C22	117.5 (2)	O34—N34—O35	123.3 (2)
C26—C21—N4	123.74 (19)	O34—N34—C34	118.16 (19)
C22—C21—N4	118.75 (19)	O35—N34—C34	118.5 (2)
C23—C22—C21	121.1 (2)	C35—C36—C31	123.71 (18)
C23—C22—H22	119.5	C35—C36—N36	117.5 (4)
C21—C22—H22	119.5	C31—C36—N36	118.7 (4)
C24—C23—C22	120.6 (2)	O36—N36—O37	123.3 (7)
C24—C23—H23	119.7	O36—N36—C36	117.9 (6)
C22—C23—H23	119.7	O37—N36—C36	118.1 (6)
C25—C24—C23	119.3 (2)	O46—N46—O47	121.8 (10)
			
C6—N1—C2—C3	−54.9 (3)	C33—C34—C35—C36	−0.9 (3)
N1—C2—C3—N4	56.9 (2)	N34—C34—C35—C36	178.29 (19)
C2—C3—N4—C21	168.24 (18)	C34—C33—C32—C31	0.7 (3)
C2—C3—N4—C5	−59.9 (2)	C34—C33—C32—N32	179.8 (6)
C21—N4—C5—C6	−168.80 (19)	O31—C31—C32—C33	175.2 (2)
C3—N4—C5—C6	60.3 (2)	C36—C31—C32—C33	−2.4 (3)
C2—N1—C6—C5	55.6 (2)	O31—C31—C32—N32	−3.8 (7)
N4—C5—C6—N1	−58.2 (2)	C36—C31—C32—N32	178.5 (6)
C5—N4—C21—C26	−4.7 (3)	C33—C32—N32—O32	−155.0 (10)
C3—N4—C21—C26	124.3 (2)	C31—C32—N32—O32	24.2 (15)
C5—N4—C21—C22	173.2 (2)	C33—C32—N32—O33	20.3 (13)
C3—N4—C21—C22	−57.8 (3)	C31—C32—N32—O33	−160.6 (8)
C26—C21—C22—C23	0.0 (3)	C35—C34—N34—O34	−177.3 (2)
N4—C21—C22—C23	−178.0 (2)	C33—C34—N34—O34	2.0 (3)
C21—C22—C23—C24	1.3 (4)	C35—C34—N34—O35	2.4 (3)
C22—C23—C24—C25	−1.2 (4)	C33—C34—N34—O35	−178.4 (2)
C22—C23—C24—O24	178.8 (2)	C34—C35—C36—C31	−1.1 (3)
C23—C24—C25—C26	−0.3 (4)	C34—C35—C36—N36	−178.7 (6)
O24—C24—C25—C26	179.8 (2)	O31—C31—C36—C35	−175.0 (2)
C22—C21—C26—C25	−1.4 (3)	C32—C31—C36—C35	2.6 (3)
N4—C21—C26—C25	176.5 (2)	O31—C31—C36—N36	2.6 (6)
C24—C25—C26—C21	1.6 (4)	C32—C31—C36—N36	−179.8 (6)
C25—C24—O24—C27	0.6 (4)	C35—C36—N36—O36	19.8 (18)
C23—C24—O24—C27	−179.4 (3)	C31—C36—N36—O36	−158.0 (16)
C32—C33—C34—C35	1.1 (3)	C35—C36—N36—O37	−151.0 (14)
C32—C33—C34—N34	−178.13 (19)	C31—C36—N36—O37	31.3 (17)

### 4-(4-Methoxyphenyl)piperazin-1-ium 2,4,6-trinitrophenolate (III) Hydrogen-bond geometry (Å, º)

**Table:**

*D*—H···*A*	*D*—H	H···*A*	*D*···*A*	*D*—H···*A*
N1—H11···O31	0.92 (3)	1.81 (3)	2.704 (3)	163 (2)
N1—H11···O37	0.92 (3)	2.56 (3)	2.982 (11)	108.7 (19)
N1—H11···O47	0.92 (3)	2.42 (3)	2.870 (15)	110.1 (19)
N1—H12···O33^i^	0.91 (3)	2.12 (3)	2.926 (6)	148 (2)
N1—H12···O43^i^	0.91 (3)	1.92 (3)	2.815 (6)	168 (2)
C22—H22···*Cg*1^ii^	0.93	2.86	3.769 (3)	164

Symmetry codes: (i) *x*−1, *y*, *z*; (ii) −*x*+1/2, *y*−1/2, −*z*+3/2.

### 4-(4-Methoxyphenyl)piperazin-1-ium 4-aminobenzoate monohydrate (IV) Crystal data

**Table:**

C_7_H_6_NO_2_^+^·C_11_H_17_N_2_O^−^·H_2_O	*Z* = 2
*M**_r_* = 347.41	*F*(000) = 372
Triclinic, *P*1	*D*_x_ = 1.301 Mg m^−^^3^
*a* = 6.2590 (7) Å	Mo *K*α radiation, λ = 0.71073 Å
*b* = 7.4549 (9) Å	Cell parameters from 3815 reflections
*c* = 19.269 (2) Å	θ = 2.9–28.0°
α = 83.28 (1)°	µ = 0.09 mm^−^^1^
β = 84.740 (1)°	*T* = 293 K
γ = 85.38 (1)°	Needle, orange
*V* = 886.94 (17) Å^3^	0.40 × 0.20 × 0.14 mm

### 4-(4-Methoxyphenyl)piperazin-1-ium 4-aminobenzoate monohydrate (IV) Data collection

**Table:**

Oxford Diffraction Xcalibur with Sapphire CCD diffractometer	3500 independent reflections
Radiation source: Enhance (Mo) X-ray Source	1923 reflections with *I* > 2σ(*I*)
Graphite monochromator	*R*_int_ = 0.031
ω scans	θ_max_ = 26.0°, θ_min_ = 3.1°
Absorption correction: multi-scan (CrysAlis RED; Oxford Diffraction, 2009)	*h* = −7→7
*T*_min_ = 0.814, *T*_max_ = 0.987	*k* = −9→9
5786 measured reflections	*l* = −23→23

### 4-(4-Methoxyphenyl)piperazin-1-ium 4-aminobenzoate monohydrate (IV) Refinement

**Table:**

Refinement on *F*^2^	Primary atom site location: dual
Least-squares matrix: full	Hydrogen site location: mixed
*R*[*F*^2^ > 2σ(*F*^2^)] = 0.069	H atoms treated by a mixture of independent and constrained refinement
*wR*(*F*^2^) = 0.187	*w* = 1/[σ^2^(*F*_o_^2^) + (0.0852*P*)^2^ + 0.1253*P*] where *P* = (*F*_o_^2^ + 2*F*_c_^2^)/3
*S* = 1.07	(Δ/σ)_max_ < 0.001
3500 reflections	Δρ_max_ = 0.24 e Å^−^^3^
240 parameters	Δρ_min_ = −0.20 e Å^−^^3^
2 restraints	

### 4-(4-Methoxyphenyl)piperazin-1-ium 4-aminobenzoate monohydrate (IV) Special details

**Table:**

Geometry. All esds (except the esd in the dihedral angle between two l.s. planes) are estimated using the full covariance matrix. The cell esds are taken into account individually in the estimation of esds in distances, angles and torsion angles; correlations between esds in cell parameters are only used when they are defined by crystal symmetry. An approximate (isotropic) treatment of cell esds is used for estimating esds involving l.s. planes.

### 4-(4-Methoxyphenyl)piperazin-1-ium 4-aminobenzoate monohydrate (IV) Fractional atomic coordinates and isotropic or equivalent isotropic displacement parameters (Å^2^)

**Table:**

	*x*	*y*	*z*	*U*_iso_*/*U*_eq_	
N1	0.2828 (4)	0.7421 (3)	0.95242 (13)	0.0450 (7)	
H11	0.2404 (10)	0.622 (3)	0.9597 (2)	0.054*	
H12	0.3170 (8)	0.7746 (7)	0.9957 (10)	0.054*	
C2	0.4741 (5)	0.7531 (5)	0.90102 (16)	0.0497 (8)	
H2A	0.5897	0.6695	0.9179	0.060*	
H2B	0.5230	0.8745	0.8959	0.060*	
C3	0.4195 (5)	0.7068 (4)	0.83065 (15)	0.0441 (8)	
H3A	0.5434	0.7229	0.7970	0.053*	
H3B	0.3885	0.5804	0.8349	0.053*	
N4	0.2361 (4)	0.8177 (3)	0.80430 (12)	0.0364 (6)	
C5	0.0500 (5)	0.8148 (4)	0.85669 (15)	0.0439 (7)	
H5A	−0.0018	0.6943	0.8637	0.053*	
H5B	−0.0648	0.8982	0.8391	0.053*	
C6	0.1042 (5)	0.8661 (4)	0.92564 (15)	0.0483 (8)	
H6A	0.1461	0.9898	0.9197	0.058*	
H6B	−0.0212	0.8590	0.9591	0.058*	
C21	0.1873 (4)	0.7910 (4)	0.73590 (14)	0.0360 (7)	
C22	0.3250 (5)	0.6885 (4)	0.69232 (16)	0.0486 (8)	
H22	0.4527	0.6338	0.7084	0.058*	
C23	0.2747 (5)	0.6671 (5)	0.62568 (17)	0.0560 (9)	
H23	0.3696	0.5990	0.5974	0.067*	
C24	0.0876 (5)	0.7445 (5)	0.60071 (16)	0.0492 (8)	
C25	−0.0491 (5)	0.8463 (5)	0.64207 (17)	0.0560 (9)	
H25	−0.1761	0.9008	0.6254	0.067*	
C26	0.0006 (5)	0.8689 (4)	0.70887 (16)	0.0524 (9)	
H26	−0.0947	0.9387	0.7364	0.063*	
O24	0.0508 (4)	0.7097 (4)	0.53404 (12)	0.0751 (8)	
C27	−0.1543 (7)	0.7604 (7)	0.5111 (2)	0.0904 (15)	
H27A	−0.2619	0.7136	0.5457	0.136*	
H27B	−0.1664	0.7123	0.4676	0.136*	
H27C	−0.1748	0.8901	0.5042	0.136*	
C31	0.2985 (4)	0.7266 (4)	1.20437 (15)	0.0377 (7)	
C32	0.1577 (5)	0.6559 (4)	1.25859 (17)	0.0499 (8)	
H32	0.0366	0.6033	1.2480	0.060*	
C33	0.1932 (5)	0.6617 (4)	1.32757 (17)	0.0543 (9)	
H33	0.0961	0.6128	1.3628	0.065*	
C34	0.3719 (5)	0.7394 (4)	1.34546 (16)	0.0501 (8)	
C35	0.5129 (5)	0.8063 (4)	1.29165 (17)	0.0497 (8)	
H35	0.6352	0.8570	1.3023	0.060*	
C36	0.4789 (5)	0.8008 (4)	1.22268 (16)	0.0439 (8)	
H36	0.5781	0.8474	1.1877	0.053*	
C37	0.2566 (5)	0.7256 (4)	1.12955 (17)	0.0477 (8)	
O31	0.3847 (4)	0.7999 (3)	1.08228 (12)	0.0614 (7)	
O32	0.0963 (4)	0.6528 (4)	1.11651 (14)	0.0866 (9)	
N34	0.4074 (6)	0.7461 (6)	1.41482 (17)	0.0767 (11)	
H341	0.312 (7)	0.732 (6)	1.447 (2)	0.092*	
H342	0.511 (7)	0.811 (6)	1.421 (2)	0.092*	
O41	0.2239 (4)	0.3712 (3)	0.96061 (13)	0.0592 (7)	
H41	0.122 (5)	0.361 (5)	0.9356 (18)	0.089*	
H42	0.329 (5)	0.307 (5)	0.943 (2)	0.089*	

### 4-(4-Methoxyphenyl)piperazin-1-ium 4-aminobenzoate monohydrate (IV) Atomic displacement parameters (Å^2^)

**Table:**

	*U*^11^	*U*^22^	*U*^33^	*U*^12^	*U*^13^	*U*^23^
N1	0.0545 (17)	0.0468 (15)	0.0367 (14)	−0.0123 (12)	−0.0095 (12)	−0.0066 (11)
C2	0.0439 (19)	0.062 (2)	0.0445 (19)	−0.0063 (15)	−0.0093 (15)	−0.0065 (15)
C3	0.0361 (17)	0.0536 (19)	0.0422 (18)	0.0048 (14)	−0.0056 (13)	−0.0072 (14)
N4	0.0364 (13)	0.0388 (13)	0.0345 (13)	−0.0009 (10)	−0.0026 (10)	−0.0074 (10)
C5	0.0384 (17)	0.0543 (19)	0.0390 (17)	0.0001 (14)	−0.0030 (13)	−0.0072 (14)
C6	0.052 (2)	0.0528 (19)	0.0394 (18)	−0.0008 (15)	−0.0025 (15)	−0.0072 (14)
C21	0.0351 (16)	0.0327 (16)	0.0404 (17)	−0.0017 (12)	−0.0040 (13)	−0.0049 (12)
C22	0.0357 (17)	0.065 (2)	0.0461 (19)	0.0099 (15)	−0.0072 (14)	−0.0152 (16)
C23	0.046 (2)	0.076 (2)	0.047 (2)	0.0101 (17)	−0.0033 (16)	−0.0237 (17)
C24	0.049 (2)	0.066 (2)	0.0344 (18)	−0.0069 (16)	−0.0061 (14)	−0.0105 (15)
C25	0.054 (2)	0.067 (2)	0.048 (2)	0.0168 (17)	−0.0173 (16)	−0.0139 (17)
C26	0.055 (2)	0.058 (2)	0.0441 (19)	0.0151 (16)	−0.0079 (15)	−0.0156 (16)
O24	0.0604 (16)	0.123 (2)	0.0470 (15)	0.0053 (15)	−0.0153 (12)	−0.0311 (14)
C27	0.075 (3)	0.148 (4)	0.055 (3)	0.002 (3)	−0.026 (2)	−0.030 (3)
C31	0.0366 (16)	0.0332 (16)	0.0448 (18)	0.0014 (13)	−0.0080 (13)	−0.0098 (13)
C32	0.0438 (18)	0.0477 (19)	0.061 (2)	−0.0079 (15)	−0.0035 (16)	−0.0152 (16)
C33	0.052 (2)	0.057 (2)	0.052 (2)	−0.0068 (17)	0.0088 (16)	−0.0039 (16)
C34	0.054 (2)	0.054 (2)	0.0415 (19)	0.0096 (16)	−0.0099 (16)	−0.0073 (15)
C35	0.0440 (19)	0.058 (2)	0.050 (2)	−0.0055 (15)	−0.0149 (16)	−0.0075 (16)
C36	0.0431 (18)	0.0435 (18)	0.0461 (19)	−0.0079 (14)	−0.0061 (14)	−0.0031 (14)
C37	0.052 (2)	0.0399 (18)	0.054 (2)	0.0030 (15)	−0.0137 (17)	−0.0144 (15)
O31	0.0741 (17)	0.0713 (16)	0.0416 (14)	−0.0104 (13)	−0.0126 (12)	−0.0088 (11)
O32	0.0760 (19)	0.116 (2)	0.080 (2)	−0.0354 (17)	−0.0310 (15)	−0.0252 (16)
N34	0.075 (3)	0.112 (3)	0.044 (2)	0.009 (2)	−0.0138 (16)	−0.0150 (19)
O41	0.0597 (16)	0.0589 (16)	0.0637 (16)	−0.0143 (12)	−0.0196 (12)	−0.0088 (12)

### 4-(4-Methoxyphenyl)piperazin-1-ium 4-aminobenzoate monohydrate (IV) Geometric parameters (Å, º)

**Table:**

N1—C6	1.483 (4)	C25—C26	1.384 (4)
N1—C2	1.484 (4)	C25—H25	0.9300
N1—H11	0.94 (2)	C26—H26	0.9300
N1—H12	0.94 (2)	O24—C27	1.405 (4)
C2—C3	1.513 (4)	C27—H27A	0.9600
C2—H2A	0.9700	C27—H27B	0.9600
C2—H2B	0.9700	C27—H27C	0.9600
C3—N4	1.454 (3)	C31—C32	1.387 (4)
C3—H3A	0.9700	C31—C36	1.387 (4)
C3—H3B	0.9700	C31—C37	1.490 (4)
N4—C21	1.420 (3)	C32—C33	1.374 (4)
N4—C5	1.470 (3)	C32—H32	0.9300
C5—C6	1.500 (4)	C33—C34	1.388 (5)
C5—H5A	0.9700	C33—H33	0.9300
C5—H5B	0.9700	C34—C35	1.373 (4)
C6—H6A	0.9700	C34—N34	1.382 (4)
C6—H6B	0.9700	C35—C36	1.371 (4)
C21—C26	1.381 (4)	C35—H35	0.9300
C21—C22	1.393 (4)	C36—H36	0.9300
C22—C23	1.380 (4)	C37—O32	1.237 (4)
C22—H22	0.9300	C37—O31	1.265 (4)
C23—C24	1.366 (4)	N34—H341	0.82 (4)
C23—H23	0.9300	N34—H342	0.87 (4)
C24—C25	1.362 (4)	O41—H41	0.850 (19)
C24—O24	1.383 (4)	O41—H42	0.856 (19)
			
C6—N1—C2	109.6 (2)	C25—C24—C23	119.2 (3)
C6—N1—H11	109.8	C25—C24—O24	124.8 (3)
C2—N1—H11	109.8	C23—C24—O24	116.0 (3)
C6—N1—H12	109.8	C24—C25—C26	120.2 (3)
C2—N1—H12	109.8	C24—C25—H25	119.9
H11—N1—H12	108.2	C26—C25—H25	119.9
N1—C2—C3	110.3 (2)	C21—C26—C25	122.0 (3)
N1—C2—H2A	109.6	C21—C26—H26	119.0
C3—C2—H2A	109.6	C25—C26—H26	119.0
N1—C2—H2B	109.6	C24—O24—C27	117.7 (3)
C3—C2—H2B	109.6	O24—C27—H27A	109.5
H2A—C2—H2B	108.1	O24—C27—H27B	109.5
N4—C3—C2	112.8 (2)	H27A—C27—H27B	109.5
N4—C3—H3A	109.0	O24—C27—H27C	109.5
C2—C3—H3A	109.0	H27A—C27—H27C	109.5
N4—C3—H3B	109.0	H27B—C27—H27C	109.5
C2—C3—H3B	109.0	C32—C31—C36	117.2 (3)
H3A—C3—H3B	107.8	C32—C31—C37	121.5 (3)
C21—N4—C3	115.5 (2)	C36—C31—C37	121.3 (3)
C21—N4—C5	114.3 (2)	C33—C32—C31	121.5 (3)
C3—N4—C5	111.3 (2)	C33—C32—H32	119.3
N4—C5—C6	112.2 (2)	C31—C32—H32	119.3
N4—C5—H5A	109.2	C32—C33—C34	121.0 (3)
C6—C5—H5A	109.2	C32—C33—H33	119.5
N4—C5—H5B	109.2	C34—C33—H33	119.5
C6—C5—H5B	109.2	C35—C34—N34	121.6 (3)
H5A—C5—H5B	107.9	C35—C34—C33	117.4 (3)
N1—C6—C5	109.8 (2)	N34—C34—C33	121.1 (3)
N1—C6—H6A	109.7	C36—C35—C34	122.0 (3)
C5—C6—H6A	109.7	C36—C35—H35	119.0
N1—C6—H6B	109.7	C34—C35—H35	119.0
C5—C6—H6B	109.7	C35—C36—C31	120.9 (3)
H6A—C6—H6B	108.2	C35—C36—H36	119.5
C26—C21—C22	116.6 (3)	C31—C36—H36	119.5
C26—C21—N4	121.0 (2)	O32—C37—O31	122.9 (3)
C22—C21—N4	122.3 (3)	O32—C37—C31	118.2 (3)
C23—C22—C21	121.0 (3)	O31—C37—C31	118.9 (3)
C23—C22—H22	119.5	C34—N34—H341	122 (3)
C21—C22—H22	119.5	C34—N34—H342	115 (3)
C24—C23—C22	120.9 (3)	H341—N34—H342	117 (4)
C24—C23—H23	119.5	H41—O41—H42	104 (4)
C22—C23—H23	119.5		
			
C6—N1—C2—C3	−57.7 (3)	C22—C21—C26—C25	0.3 (5)
N1—C2—C3—N4	54.8 (3)	N4—C21—C26—C25	179.3 (3)
C2—C3—N4—C21	175.1 (2)	C24—C25—C26—C21	0.1 (5)
C2—C3—N4—C5	−52.3 (3)	C25—C24—O24—C27	−9.6 (5)
C21—N4—C5—C6	−173.0 (2)	C23—C24—O24—C27	169.5 (4)
C3—N4—C5—C6	53.8 (3)	C36—C31—C32—C33	1.1 (4)
C2—N1—C6—C5	59.3 (3)	C37—C31—C32—C33	−178.0 (3)
N4—C5—C6—N1	−57.6 (3)	C31—C32—C33—C34	0.2 (5)
C3—N4—C21—C26	170.9 (3)	C32—C33—C34—C35	−1.3 (5)
C5—N4—C21—C26	39.7 (4)	C32—C33—C34—N34	179.7 (3)
C3—N4—C21—C22	−10.2 (4)	N34—C34—C35—C36	−179.8 (3)
C5—N4—C21—C22	−141.4 (3)	C33—C34—C35—C36	1.2 (5)
C26—C21—C22—C23	−0.1 (5)	C34—C35—C36—C31	0.0 (5)
N4—C21—C22—C23	−179.0 (3)	C32—C31—C36—C35	−1.2 (4)
C21—C22—C23—C24	−0.5 (5)	C37—C31—C36—C35	177.9 (3)
C22—C23—C24—C25	1.0 (5)	C32—C31—C37—O32	−2.9 (4)
C22—C23—C24—O24	−178.2 (3)	C36—C31—C37—O32	178.0 (3)
C23—C24—C25—C26	−0.8 (5)	C32—C31—C37—O31	176.5 (3)
O24—C24—C25—C26	178.3 (3)	C36—C31—C37—O31	−2.6 (4)

### 4-(4-Methoxyphenyl)piperazin-1-ium 4-aminobenzoate monohydrate (IV) Hydrogen-bond geometry (Å, º)

**Table:**

*D*—H···*A*	*D*—H	H···*A*	*D*···*A*	*D*—H···*A*
N1—H11···O41	0.95 (2)	1.88 (2)	2.803 (3)	165 (1)
N1—H12···O31	0.94 (1)	1.79 (2)	2.728 (3)	171 (1)
O41—H41···O32^i^	0.85 (3)	1.78 (3)	2.631 (4)	178 (4)
O41—H42···O31^ii^	0.85 (3)	1.95 (3)	2.772 (3)	164 (3)
N34—H341···O24^iii^	0.82 (4)	2.23 (4)	3.057 (4)	177 (4)
C22—H22···*Cg*2^i^	0.93	2.93	3.666 (3)	137
C26—H26···*Cg*2^iv^	0.93	2.77	3.531 (3)	139

Symmetry codes: (i) −*x*, −*y*+1, −*z*+2; (ii) −*x*+1, −*y*+1, −*z*+2; (iii) *x*, *y*, *z*+1; (iv) −*x*, −*y*+2, −*z*+2.
